# Supplementary material for: Automated and unbiased discrimination of ALS from control tissue at single cell resolution
Source: Brain Pathol. 2021 Feb 11;31(4):e12937. doi: 10.1111/bpa.12937 (PMC8412073; doi:10.1111/bpa.12937)
Supplement: Supplementary file 1 — Supplementary Material [file BPA-31-e12937-s002.docx]

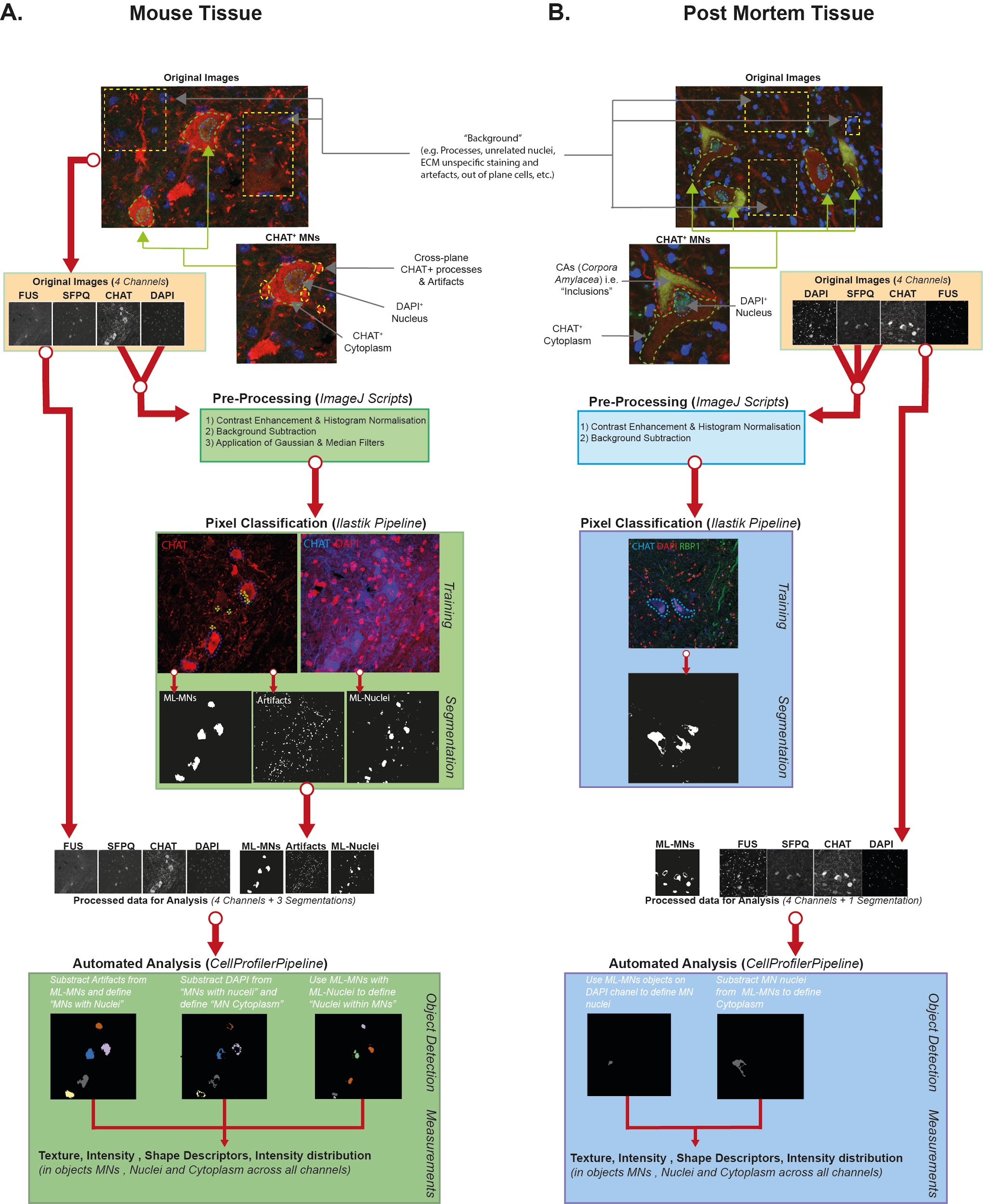


**Supplementary Figure 1 |** (**A**) Diagram representing the image processing workflow for mouse tissue immunolabeled for FUS, SFPQ, ChAT and counterstained with DAPI. To facilitate the MN segmentation, we first applied contrast enhancement, background correction and application of gaussian blur and median filters to ChAT stained images using ImageJ [(Schneider *et al.*, 2012)](https://paperpile.com/c/dbswo3/orcJn). Next we used a subset of these preprocessed ChAT images to train a pixel classification algorithm in Ilastik [(Berg *et al.*, 2019*a*)](https://paperpile.com/c/dbswo3/DRfYk) for automated identification of artifacts and MNs. In parallel, automated nuclear segmentation was trained in Ilastik on randomly selected subsets of overlaid ChAT and DAPI images. Finally, all generated segmentations were added to the original dataset as additional channels, and used to identify the cytoplasm of MNs and perform automated densitometry and morphometric measurements in each compartment (cytoplasm, nucleus, whole MNs) in CellProfiler [(Carpenter *et al.*, 2006)](https://paperpile.com/c/dbswo3/d9g8y).(**B**) Diagram representing the image processing workflow for human post-mortem tissue immunolabeled for ChAT, FUS or SFPQ, and counterstained with DAPI. Individual channels were preprocessed in ImageJ for contrast enhancement and histogram equalisation. Next automated MN segmentation was trained using a subset of the original images using IIastik based on the three channels (SFPQ, DAPI, ChAT). Next DAPI channel and the masked MNs were provided to CellProfiler for automated segmentation of nuclei and cytoplasm followed by automatic acquisition of single-cell measurements in each compartment.


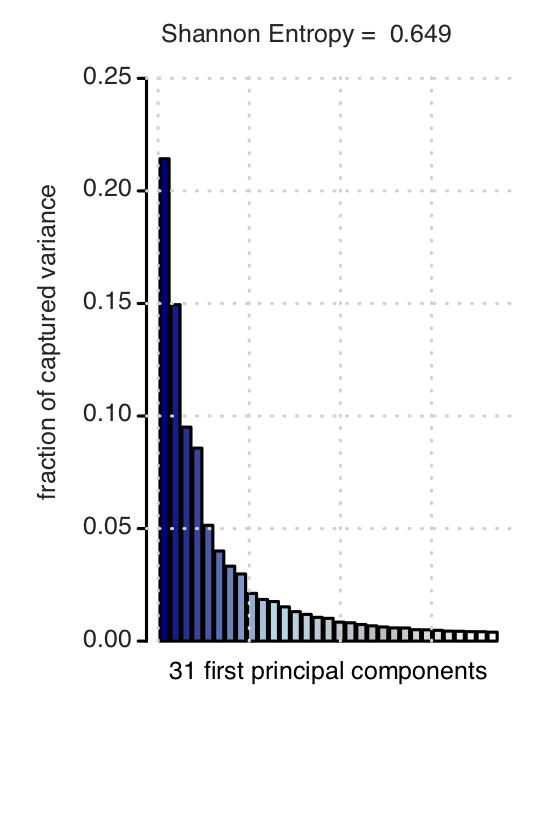


**Supplementary Figure 2 |** Fraction of explained variance captured by the first 31 principal components that captures 90% of the signal. Shannon Entropy of 0.65 indicates that the information in the data is well distributed among the principal components.


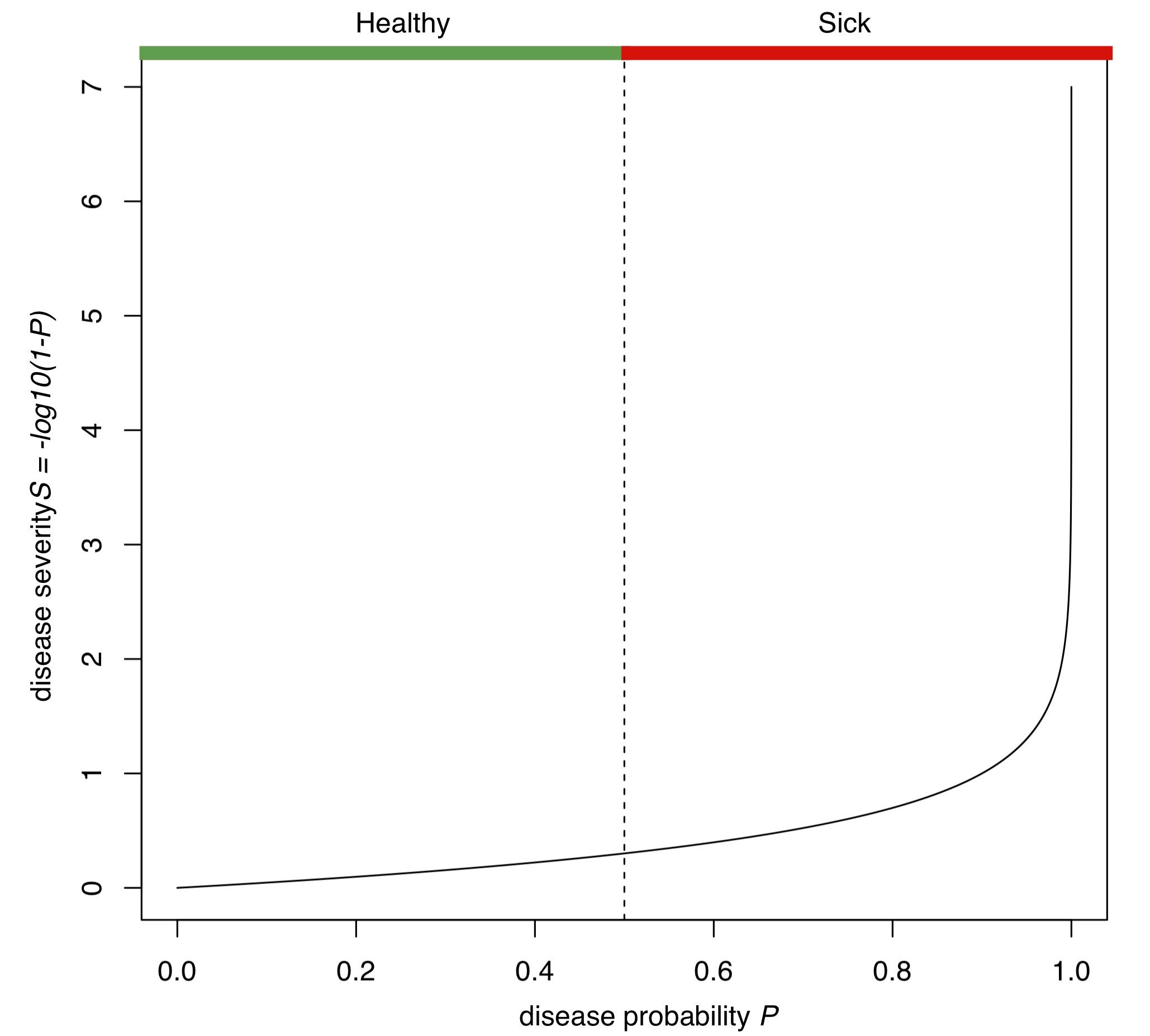


**Supplementary Figure 3 |** Comparison between disease probability P and disease severity S scores showing how similarly high disease probability can exhibit large differences in disease severity.


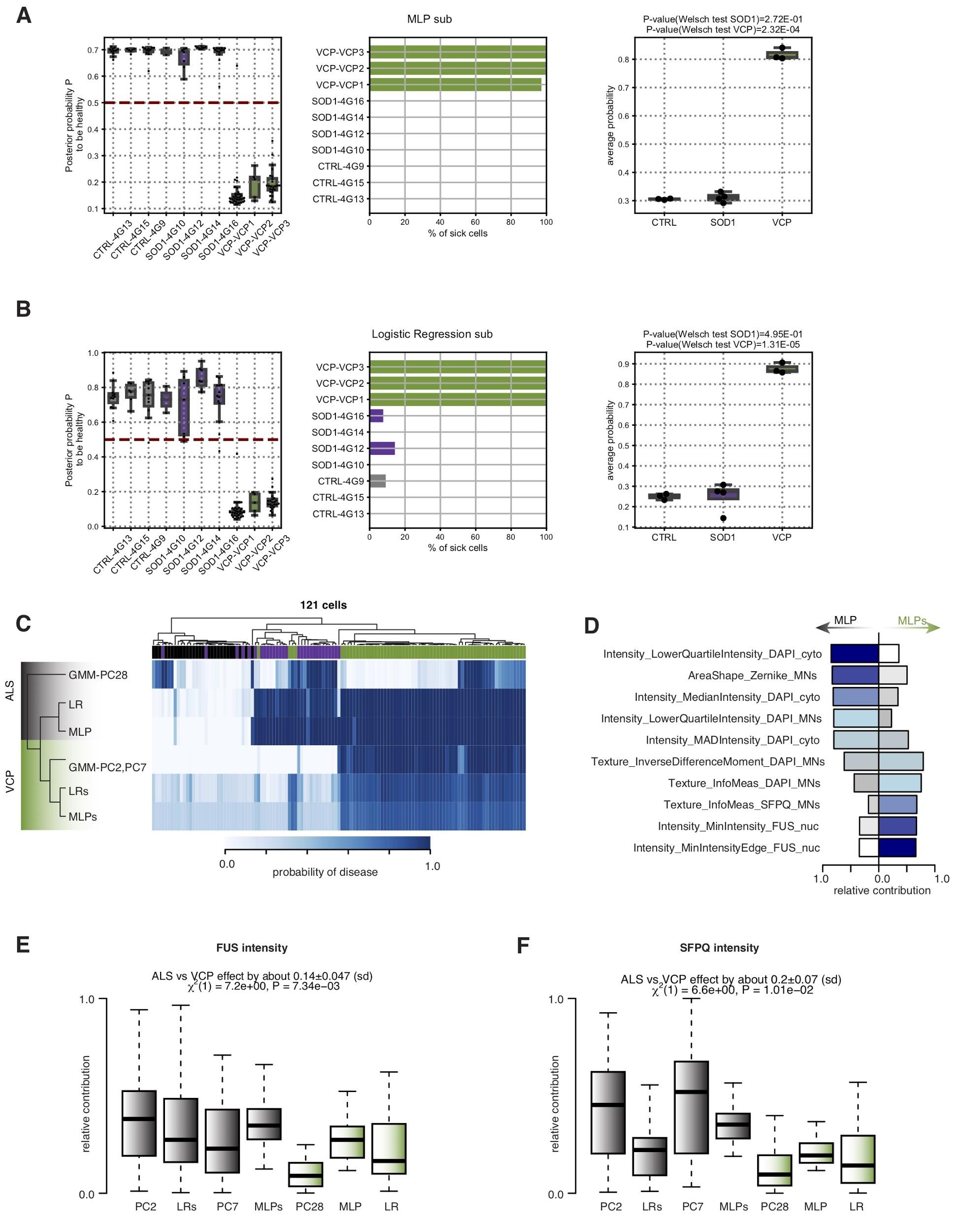


**Supplementary Figure 4 |** (**A**, **B**) MNs predicted probability distribution (*left*), per-animal percentage of sick cells (*centre*), and per-animal disease probability *(right)* as obtained by LR classifier (**A**) and MLP classifier (**B**) trained on data censored for SOD1-mutant cells. (**C**) Heatmap showing the predicted disease probability for the 121 cells. Classifiers are hierarchically clustered using average alog on euclidean distances between disease probability profiles across the 121 cells. Green = *vcpALS* classifiers. Grey = *comALS* classifiers. (**D**) Barplots showing the relative contribution of the top five measurements in MLP and MLPs i.e. *comALS* versus *vcpALS* classifiers. Zernike moments either in the nucleus or the whole MNs contribute largely to ALS but not VCP classifier. Bars are color-coded according to the ranking in contribution for the given classifier, from dark blue to white for high to low ranking. (**E,F**) Boxplots showing the relative contribution of FUS (**E**) and SFPQ (**F**) intensity related measurements in *vcpALS* versus *comALS* classifiers. Linear mixed effects analysis of the relationship between the type of classifiers (*comALS* versus *vcpALS*) and the relative contribution of the measurement categories to account for idiosyncratic variation due to classifiers. Data shown as box plots in which the centre line is the median, limits are the interquartile range and whiskers are the minimum and maximum. Green = *vcpALS* classifiers; grey = *comALS* classifiers.


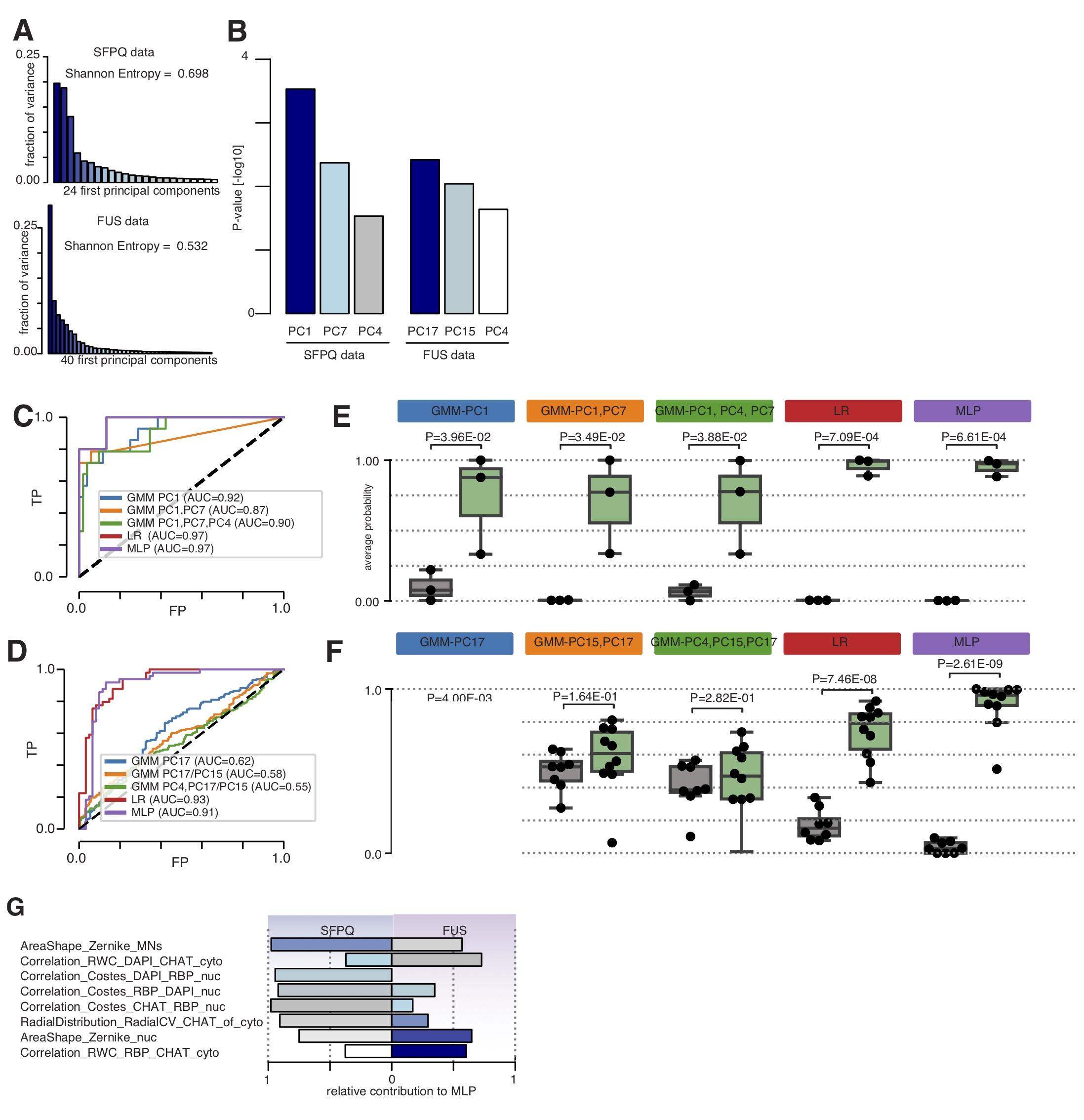


**Supplementary Figure 5 |** (**A**) Fraction of explained variance captured by the first 24 and 40 principal components that captures 90% of the signal in SFPQ and FUS data respectively. (**B**) Barplots showing the association between principal components and ALS in SFPQ (*left)* and FUS (*right*) data. Linear mixed effects analysis of the relationship between ALS phenotype and each of the 24 and 40 first principal components to account for idiosyncratic variation due to individuals shows significant association of PC1, PC4 and PC7 and ALS in SFPQ data, and association between PC4, PC15 and PC17 with ALS in FUS data. (**C**,**D**) Performance analysis of each classifier in SFPQ data (**C**) or FUS data (**D**) in their ability to discriminate sALS MNs from healthy MNs using receiver operating characteristic (ROC) curves and aurea under the curves (AUC). (**E**, **F**) The ability for each clustering algorithm to detect sALS effect is assessed by comparing the disease probabilities of sALS group with those of the control group. The disease probability of each individual is obtained by using the mean probabilities of its cells to be sick according to individual classifiers in SFPQ data (**B**) or FUS data (**F**). Data shown as box plots in which the centre line is the median, limits are the interquartile range and whiskers are the minimum and maximum. Dots are the individual disease profile. *P*-values obtained from Welch’s t test. (**G**) Barplots showing the relative contribution of the top five measurements in MLP in either SFPQ or FUS post-mortem tissue data. Zernike moments either in the nucleus or the whole MNs contribute largely to both MLP classifiers. Bars are color-coded according to the ranking in contribution for the given classifier, from dark blue to white for high to low ranking.

**TABLES**

| **imageID** | **mutation** | **RBP1** | **RBP2** | **animal** | **origin** | **Lab** | **pairs** |
| --- | --- | --- | --- | --- | --- | --- | --- |
| **4G10D1** | SOD1 | SFPQ | FUS | 4G10 | Lab_2 | Lab_2 | SFPQ_FUS |
| **4G10D2** | SOD1 | SFPQ | FUS | 4G10 | Lab_2 | Lab_2 | SFPQ_FUS |
| **4G12D1** | SOD1 | SFPQ | FUS | 4G12 | Lab_2 | Lab_2 | SFPQ_FUS |
| **4G12D2** | SOD1 | SFPQ | FUS | 4G12 | Lab_2 | Lab_2 | SFPQ_FUS |
| **4G13D1** | CTRL | SFPQ | FUS | 4G13 | Lab_2 | Lab_2 | SFPQ_FUS |
| **4G13D2** | CTRL | SFPQ | FUS | 4G13 | Lab_2 | Lab_2 | SFPQ_FUS |
| **4G13D3** | CTRL | SFPQ | FUS | 4G13 | Lab_2 | Lab_2 | SFPQ_FUS |
| **4G14D1** | SOD1 | SFPQ | FUS | 4G14 | Lab_2 | Lab_2 | SFPQ_FUS |
| **4G14D2** | SOD1 | SFPQ | FUS | 4G14 | Lab_2 | Lab_2 | SFPQ_FUS |
| **4G14D3** | SOD1 | SFPQ | FUS | 4G14 | Lab_2 | Lab_2 | SFPQ_FUS |
| **4G15D1** | CTRL | SFPQ | FUS | 4G15 | Lab_2 | Lab_2 | SFPQ_FUS |
| **4G15D2** | CTRL | SFPQ | FUS | 4G15 | Lab_2 | Lab_2 | SFPQ_FUS |
| **4G16D1** | SOD1 | SFPQ | FUS | 4G16 | Lab_2 | Lab_2 | SFPQ_FUS |
| **4G16D2** | SOD1 | SFPQ | FUS | 4G16 | Lab_2 | Lab_2 | SFPQ_FUS |
| **4G16D3** | SOD1 | SFPQ | FUS | 4G16 | Lab_2 | Lab_2 | SFPQ_FUS |
| **4G9D1** | CTRL | SFPQ | FUS | 4G9 | Lab_2 | Lab_2 | SFPQ_FUS |
| **4G9D2** | CTRL | SFPQ | FUS | 4G9 | Lab_2 | Lab_2 | SFPQ_FUS |
| **4G9D3** | CTRL | SFPQ | FUS | 4G9 | Lab_2 | Lab_2 | SFPQ_FUS |
| **VCP1AFITC** | VCP | SFPQ | FUS | VCP1 | Lab_1 | Lab_1 | SFPQ_FUS |
| **VCP1BCY5** | VCP | SFPQ | FUS | VCP1 | Lab_1 | Lab_1 | SFPQ_FUS |
| **VCP1BFITC** | VCP | SFPQ | FUS | VCP1 | Lab_1 | Lab_1 | SFPQ_FUS |
| **VCP1CFITC** | VCP | SFPQ | FUS | VCP1 | Lab_1 | Lab_1 | SFPQ_FUS |
| **VCP1DFITC** | VCP | SFPQ | FUS | VCP1 | Lab_1 | Lab_1 | SFPQ_FUS |
| **VCP1EFITC** | VCP | SFPQ | FUS | VCP1 | Lab_1 | Lab_1 | SFPQ_FUS |
| **VCP2A** | VCP | SFPQ | FUS | VCP2 | Lab_1 | Lab_1 | SFPQ_FUS |
| **VCP2AMODIFIED** | VCP | SFPQ | FUS | VCP2 | Lab_1 | Lab_1 | SFPQ_FUS |
| **VCP2BFITIC** | VCP | SFPQ | FUS | VCP2 | Lab_1 | Lab_1 | SFPQ_FUS |
| **VCP2BMODIF** | VCP | SFPQ | FUS | VCP2 | Lab_1 | Lab_1 | SFPQ_FUS |
| **VCP2CFITIC** | VCP | SFPQ | FUS | VCP2 | Lab_1 | Lab_1 | SFPQ_FUS |
| **VCP2DFITC** | VCP | SFPQ | FUS | VCP2 | Lab_1 | Lab_1 | SFPQ_FUS |
| **VCP2EFITC** | VCP | SFPQ | FUS | VCP2 | Lab_1 | Lab_1 | SFPQ_FUS |
| **VCP2FFITC** | VCP | SFPQ | FUS | VCP2 | Lab_1 | Lab_1 | SFPQ_FUS |
| **VCP3A** | VCP | SFPQ | FUS | VCP3 | Lab_1 | Lab_1 | SFPQ_FUS |
| **VCP3B** | VCP | SFPQ | FUS | VCP3 | Lab_1 | Lab_1 | SFPQ_FUS |
| **VCP3C** | VCP | SFPQ | FUS | VCP3 | Lab_1 | Lab_1 | SFPQ_FUS |
| **VCP3D** | VCP | SFPQ | FUS | VCP3 | Lab_1 | Lab_1 | SFPQ_FUS |
| **VCP3E** | VCP | SFPQ | FUS | VCP3 | Lab_1 | Lab_1 | SFPQ_FUS |
| **VCP3F** | VCP | SFPQ | FUS | VCP3 | Lab_1 | Lab_1 | SFPQ_FUS |

**Table S1 |** List of images used for FUS and SFPQ cellular localisation in [(Luisier *et al.*, 2018; Tyzack *et al.*, 2019)](https://paperpile.com/c/dbswo3/0HdLy+vHVXw); mouse data.

| **Batch** | **ID** | **ImageID** | **RBP** | **Group** | **imageID** | **frame** | **round** | **patient** | **rbp1** |
| --- | --- | --- | --- | --- | --- | --- | --- | --- | --- |
| **1TG** | 1TG1 | TG1AL1-ChAT.tif | FUS | CTRL | 1TG1AL1 | AL1 | AL | 1TG1 | FUS |
| **1TG** | 1TG1 | TG1AL2-ChAT.tif | FUS | CTRL | 1TG1AL2 | AL2 | AL | 1TG1 | FUS |
| **1TG** | 1TG1 | TG1AL3-ChAT.tif | FUS | CTRL | 1TG1AL3 | AL3 | AL | 1TG1 | FUS |
| **1TG** | 1TG1 | TG1AL4-ChAT.tif | FUS | CTRL | 1TG1AL4 | AL4 | AL | 1TG1 | FUS |
| **1TG** | 1TG1 | TG1AL5-ChAT.tif | FUS | CTRL | 1TG1AL5 | AL5 | AL | 1TG1 | FUS |
| **1TG** | 1TG1 | TG1AM1-ChAT.tif | FUS | CTRL | 1TG1AM1 | AM1 | AM | 1TG1 | FUS |
| **1TG** | 1TG1 | TG1AM2-ChAT.tif | FUS | CTRL | 1TG1AM2 | AM2 | AM | 1TG1 | FUS |
| **1TG** | 1TG1 | TG1AM3-ChAT.tif | FUS | CTRL | 1TG1AM3 | AM3 | AM | 1TG1 | FUS |
| **1TG** | 1TG1 | TG1AM4-ChAT.tif | FUS | CTRL | 1TG1AM4 | AM4 | AM | 1TG1 | FUS |
| **1TG** | 1TG1 | TG1AR1-ChAT.tif | FUS | CTRL | 1TG1AR1 | AR1 | AR | 1TG1 | FUS |
| **1TG** | 1TG1 | TG1AR2-ChAT.tif | FUS | CTRL | 1TG1AR2 | AR2 | AR | 1TG1 | FUS |
| **1TG** | 1TG1 | TG1AR3-ChAT.tif | FUS | CTRL | 1TG1AR3 | AR3 | AR | 1TG1 | FUS |
| **1TG** | 1TG1 | TG1AR4-ChAT.tif | FUS | CTRL | 1TG1AR4 | AR4 | AR | 1TG1 | FUS |
| **1TG** | 1TG2 | TG2A-ChAT.tif | FUS | CTRL | 1TG2A | A | A | 1TG2 | FUS |
| **1TG** | 1TG2 | TG2Al1-ChAT.tif | FUS | CTRL | 1TG2Al1 | Al1 | Al | 1TG2 | FUS |
| **1TG** | 1TG2 | TG2AL3-ChAT.tif | FUS | CTRL | 1TG2AL3 | AL3 | AL | 1TG2 | FUS |
| **1TG** | 1TG2 | TG2AL4-ChAT.tif | FUS | CTRL | 1TG2AL4 | AL4 | AL | 1TG2 | FUS |
| **1TG** | 1TG2 | TG2AM1-ChAT.tif | FUS | CTRL | 1TG2AM1 | AM1 | AM | 1TG2 | FUS |
| **1TG** | 1TG2 | TG2AM2-ChAT.tif | FUS | CTRL | 1TG2AM2 | AM2 | AM | 1TG2 | FUS |
| **1TG** | 1TG2 | TG2AM23-ChAT.tif | FUS | CTRL | 1TG2AM23 | AM23 | AM | 1TG2 | FUS |
| **1TG** | 1TG2 | TG2AM4-ChAT.tif | FUS | CTRL | 1TG2AM4 | AM4 | AM | 1TG2 | FUS |
| **1TG** | 1TG2 | TG2AM5-ChAT.tif | FUS | CTRL | 1TG2AM5 | AM5 | AM | 1TG2 | FUS |
| **1TG** | 1TG2 | TG2AR1-ChAT.tif | FUS | CTRL | 1TG2AR1 | AR1 | AR | 1TG2 | FUS |
| **1TG** | 1TG2 | TG2AR2-ChAT.tif | FUS | CTRL | 1TG2AR2 | AR2 | AR | 1TG2 | FUS |
| **1TG** | 1TG2 | TG2AR3-ChAT.tif | FUS | CTRL | 1TG2AR3 | AR3 | AR | 1TG2 | FUS |
| **1TG** | 1TG2 | TG2AR4-ChAT.tif | FUS | CTRL | 1TG2AR4 | AR4 | AR | 1TG2 | FUS |
| **1TG** | 1TG2 | TG2AR5-ChAT.tif | FUS | CTRL | 1TG2AR5 | AR5 | AR | 1TG2 | FUS |
| **1TG** | 1TG2 | TG2AR6-ChAT.tif | FUS | CTRL | 1TG2AR6 | AR6 | AR | 1TG2 | FUS |
| **1TG** | 1TG2 | TG2AR7-ChAT.tif | FUS | CTRL | 1TG2AR7 | AR7 | AR | 1TG2 | FUS |
| **1TG** | 1TG2 | TG2AR8-ChAT.tif | FUS | CTRL | 1TG2AR8 | AR8 | AR | 1TG2 | FUS |
| **1TG** | 1TG3 | TG3AL1-ChAT.tif | FUS | sALS | 1TG3AL1 | AL1 | AL | 1TG3 | FUS |
| **1TG** | 1TG3 | TG3AL2-ChAT.tif | FUS | sALS | 1TG3AL2 | AL2 | AL | 1TG3 | FUS |
| **1TG** | 1TG3 | TG3AL3-ChAT.tif | FUS | sALS | 1TG3AL3 | AL3 | AL | 1TG3 | FUS |
| **1TG** | 1TG3 | TG3AL4-ChAT.tif | FUS | sALS | 1TG3AL4 | AL4 | AL | 1TG3 | FUS |
| **1TG** | 1TG3 | TG3Am1-ChAT.tif | FUS | sALS | 1TG3Am1 | Am1 | Am | 1TG3 | FUS |
| **1TG** | 1TG3 | TG3AM2-ChAT.tif | FUS | sALS | 1TG3AM2 | AM2 | AM | 1TG3 | FUS |
| **1TG** | 1TG3 | TG3AM3-ChAT.tif | FUS | sALS | 1TG3AM3 | AM3 | AM | 1TG3 | FUS |
| **1TG** | 1TG3 | TG3AM4-ChAT.tif | FUS | sALS | 1TG3AM4 | AM4 | AM | 1TG3 | FUS |
| **1TG** | 1TG3 | TG3AM5-ChAT.tif | FUS | sALS | 1TG3AM5 | AM5 | AM | 1TG3 | FUS |
| **1TG** | 1TG3 | TG3AM6-ChAT.tif | FUS | sALS | 1TG3AM6 | AM6 | AM | 1TG3 | FUS |
| **1TG** | 1TG3 | TG3AM7-ChAT.tif | FUS | sALS | 1TG3AM7 | AM7 | AM | 1TG3 | FUS |
| **1TG** | 1TG3 | TG3AR1-ChAT.tif | FUS | sALS | 1TG3AR1 | AR1 | AR | 1TG3 | FUS |
| **1TG** | 1TG3 | TG3AR2-ChAT.tif | FUS | sALS | 1TG3AR2 | AR2 | AR | 1TG3 | FUS |
| **1TG** | 1TG3 | TG3AR3-ChAT.tif | FUS | sALS | 1TG3AR3 | AR3 | AR | 1TG3 | FUS |
| **1TG** | 1TG3 | TG3AR4-ChAT.tif | FUS | sALS | 1TG3AR4 | AR4 | AR | 1TG3 | FUS |
| **1TG** | 1TG4 | TG4L1-ChAT.tif | FUS | sALS | 1TG4L1 | L1 | L | 1TG4 | FUS |
| **1TG** | 1TG4 | TG4L2-ChAT.tif | FUS | sALS | 1TG4L2 | L2 | L | 1TG4 | FUS |
| **1TG** | 1TG4 | TG4L3-ChAT.tif | FUS | sALS | 1TG4L3 | L3 | L | 1TG4 | FUS |
| **1TG** | 1TG4 | TG4L4-ChAT.tif | FUS | sALS | 1TG4L4 | L4 | L | 1TG4 | FUS |
| **1TG** | 1TG4 | TG4M1-ChAT.tif | FUS | sALS | 1TG4M1 | M1 | M | 1TG4 | FUS |
| **1TG** | 1TG4 | TG4M2-ChAT.tif | FUS | sALS | 1TG4M2 | M2 | M | 1TG4 | FUS |
| **1TG** | 1TG4 | TG4M3-ChAT.tif | FUS | sALS | 1TG4M3 | M3 | M | 1TG4 | FUS |
| **1TG** | 1TG4 | TG4M4-ChAT.tif | FUS | sALS | 1TG4M4 | M4 | M | 1TG4 | FUS |
| **1TG** | 1TG4 | TG4M5-ChAT.tif | FUS | sALS | 1TG4M5 | M5 | M | 1TG4 | FUS |
| **1TG** | 1TG4 | TG4R1-ChAT.tif | FUS | sALS | 1TG4R1 | R1 | R | 1TG4 | FUS |
| **1TG** | 1TG4 | TG4R2-ChAT.tif | FUS | sALS | 1TG4R2 | R2 | R | 1TG4 | FUS |
| **1TG** | 1TG4 | TG4R3-ChAT.tif | FUS | sALS | 1TG4R3 | R3 | R | 1TG4 | FUS |
| **1TG** | 1TG4 | TG4R4-ChAT.tif | FUS | sALS | 1TG4R4 | R4 | R | 1TG4 | FUS |
| **1TG** | 1TG4 | TG4R5-ChAT.tif | FUS | sALS | 1TG4R5 | R5 | R | 1TG4 | FUS |
| **1TG** | 1TG5 | TG5Al1-ChAT.tif | FUS | sALS | 1TG5Al1 | Al1 | Al | 1TG5 | FUS |
| **1TG** | 1TG5 | TG5AL2-ChAT.tif | FUS | sALS | 1TG5AL2 | AL2 | AL | 1TG5 | FUS |
| **1TG** | 1TG5 | TG5AM1-ChAT.tif | FUS | sALS | 1TG5AM1 | AM1 | AM | 1TG5 | FUS |
| **1TG** | 1TG5 | TG5AM2-ChAT.tif | FUS | sALS | 1TG5AM2 | AM2 | AM | 1TG5 | FUS |
| **1TG** | 1TG5 | TG5AM3-ChAT.tif | FUS | sALS | 1TG5AM3 | AM3 | AM | 1TG5 | FUS |
| **1TG** | 1TG5 | TG5AM4-ChAT.tif | FUS | sALS | 1TG5AM4 | AM4 | AM | 1TG5 | FUS |
| **1TG** | 1TG5 | TG5AR1-ChAT.tif | FUS | sALS | 1TG5AR1 | AR1 | AR | 1TG5 | FUS |
| **1TG** | 1TG5 | TG5AR2-ChAT.tif | FUS | sALS | 1TG5AR2 | AR2 | AR | 1TG5 | FUS |
| **1TG** | 1TG5 | TG5AR3-ChAT.tif | FUS | sALS | 1TG5AR3 | AR3 | AR | 1TG5 | FUS |
| **1TG** | 1TG5 | TG5AR4-ChAT.tif | FUS | sALS | 1TG5AR4 | AR4 | AR | 1TG5 | FUS |
| **2TG** | 2TG4 | 2TG4A1-ChAT.tif | FUS | sALS | 2TG4A1 | A1 | A | 2TG4 | FUS |
| **2TG** | 2TG4 | 2TG4A10-ChAT.tif | FUS | sALS | 2TG4A10 | A10 | A | 2TG4 | FUS |
| **2TG** | 2TG4 | 2TG4A2-ChAT.tif | FUS | sALS | 2TG4A2 | A2 | A | 2TG4 | FUS |
| **2TG** | 2TG4 | 2TG4A3-ChAT.tif | FUS | sALS | 2TG4A3 | A3 | A | 2TG4 | FUS |
| **2TG** | 2TG4 | 2TG4A4-ChAT.tif | FUS | sALS | 2TG4A4 | A4 | A | 2TG4 | FUS |
| **2TG** | 2TG4 | 2TG4A5-ChAT.tif | FUS | sALS | 2TG4A5 | A5 | A | 2TG4 | FUS |
| **2TG** | 2TG4 | 2TG4A6-ChAT.tif | FUS | sALS | 2TG4A6 | A6 | A | 2TG4 | FUS |
| **2TG** | 2TG4 | 2TG4A7-ChAT.tif | FUS | sALS | 2TG4A7 | A7 | A | 2TG4 | FUS |
| **2TG** | 2TG4 | 2TG4A8-ChAT.tif | FUS | sALS | 2TG4A8 | A8 | A | 2TG4 | FUS |
| **2TG** | 2TG4 | 2TG4A9-ChAT.tif | FUS | sALS | 2TG4A9 | A9 | A | 2TG4 | FUS |
| **2TG** | 2TG5 | 2TG5A1-ChAT.tif | FUS | sALS | 2TG5A1 | A1 | A | 2TG5 | FUS |
| **2TG** | 2TG5 | 2TG5A2-ChAT.tif | FUS | sALS | 2TG5A2 | A2 | A | 2TG5 | FUS |
| **2TG** | 2TG5 | 2TG5A3-ChAT.tif | FUS | sALS | 2TG5A3 | A3 | A | 2TG5 | FUS |
| **2TG** | 2TG5 | 2TG5A4-ChAT.tif | FUS | sALS | 2TG5A4 | A4 | A | 2TG5 | FUS |
| **2TG** | 2TG5 | 2TG5A5-ChAT.tif | FUS | sALS | 2TG5A5 | A5 | A | 2TG5 | FUS |
| **2TG** | 2TG5 | 2TG5A6-ChAT.tif | FUS | sALS | 2TG5A6 | A6 | A | 2TG5 | FUS |
| **2TG** | 2TG5 | 2TG5A7-ChAT.tif | FUS | sALS | 2TG5A7 | A7 | A | 2TG5 | FUS |
| **2TG** | 2TG6 | 2TG6A1-ChAT.tif | FUS | sALS | 2TG6A1 | A1 | A | 2TG6 | FUS |
| **2TG** | 2TG6 | 2TG6A2-ChAT.tif | FUS | sALS | 2TG6A2 | A2 | A | 2TG6 | FUS |
| **2TG** | 2TG6 | 2TG6A3-ChAT.tif | FUS | sALS | 2TG6A3 | A3 | A | 2TG6 | FUS |
| **2TG** | 2TG6 | 2TG6A4-ChAT.tif | FUS | sALS | 2TG6A4 | A4 | A | 2TG6 | FUS |
| **2TG** | 2TG6 | 2TG6A5-ChAT.tif | FUS | sALS | 2TG6A5 | A5 | A | 2TG6 | FUS |
| **2TG** | 2TG6 | 2TG6A6-ChAT.tif | FUS | sALS | 2TG6A6 | A6 | A | 2TG6 | FUS |
| **2TG** | 2TG6 | 2TG6A7-ChAT.tif | FUS | sALS | 2TG6A7 | A7 | A | 2TG6 | FUS |
| **2TG** | 2TG6 | 2TG6A8-ChAT.tif | FUS | sALS | 2TG6A8 | A8 | A | 2TG6 | FUS |
| **3TG** | 3TG1 | 3TG1A1-ChAT.tif | FUS | CTRL | 3TG1A1 | A1 | A | 3TG1 | FUS |
| **3TG** | 3TG1 | 3TG1A2-ChAT.tif | FUS | CTRL | 3TG1A2 | A2 | A | 3TG1 | FUS |
| **3TG** | 3TG1 | 3TG1A3-ChAT.tif | FUS | CTRL | 3TG1A3 | A3 | A | 3TG1 | FUS |
| **3TG** | 3TG1 | 3TG1A4-ChAT.tif | FUS | CTRL | 3TG1A4 | A4 | A | 3TG1 | FUS |
| **3TG** | 3TG1 | 3TG1A5-ChAT.tif | FUS | CTRL | 3TG1A5 | A5 | A | 3TG1 | FUS |
| **3TG** | 3TG1 | 3TG1A6-ChAT.tif | FUS | CTRL | 3TG1A6 | A6 | A | 3TG1 | FUS |
| **3TG** | 3TG2 | 3TG2A1-ChAT.tif | FUS | CTRL | 3TG2A1 | A1 | A | 3TG2 | FUS |
| **3TG** | 3TG2 | 3TG2A2-ChAT.tif | FUS | CTRL | 3TG2A2 | A2 | A | 3TG2 | FUS |
| **3TG** | 3TG2 | 3TG2A3-ChAT.tif | FUS | CTRL | 3TG2A3 | A3 | A | 3TG2 | FUS |
| **3TG** | 3TG2 | 3TG2A4-ChAT.tif | FUS | CTRL | 3TG2A4 | A4 | A | 3TG2 | FUS |
| **3TG** | 3TG2 | 3TG2A5-ChAT.tif | FUS | CTRL | 3TG2A5 | A5 | A | 3TG2 | FUS |
| **3TG** | 3TG2 | 3TG2A6-ChAT.tif | FUS | CTRL | 3TG2A6 | A6 | A | 3TG2 | FUS |
| **3TG** | 3TG2 | 3TG2A7-ChAT.tif | FUS | CTRL | 3TG2A7 | A7 | A | 3TG2 | FUS |
| **3TG** | 3TG3 | 3TG3A1-ChAT.tif | FUS | CTRL | 3TG3A1 | A1 | A | 3TG3 | FUS |
| **3TG** | 3TG3 | 3TG3A2-ChAT.tif | FUS | CTRL | 3TG3A2 | A2 | A | 3TG3 | FUS |
| **3TG** | 3TG3 | 3TG3A3-ChAT.tif | FUS | CTRL | 3TG3A3 | A3 | A | 3TG3 | FUS |
| **3TG** | 3TG3 | 3TG3A4-ChAT.tif | FUS | CTRL | 3TG3A4 | A4 | A | 3TG3 | FUS |
| **3TG** | 3TG3 | 3TG3A5-ChAT.tif | FUS | CTRL | 3TG3A5 | A5 | A | 3TG3 | FUS |
| **3TG** | 3TG3 | 3TG3A6-ChAT.tif | FUS | CTRL | 3TG3A6 | A6 | A | 3TG3 | FUS |
| **3TG** | 3TG3 | 3TG3A7-ChAT.tif | FUS | CTRL | 3TG3A7 | A7 | A | 3TG3 | FUS |
| **3TG** | 3TG3 | 3TG3A8 | FUS | CTRL | 3TG3A8 | A8 | A | 3TG3 | FUS |
| **3TG** | 3TG4 | 3TG4A1-ChAT.tif | FUS | sALS | 3TG4A1 | A1 | A | 3TG4 | FUS |
| **3TG** | 3TG4 | 3TG4A2-ChAT.tif | FUS | sALS | 3TG4A2 | A2 | A | 3TG4 | FUS |
| **3TG** | 3TG4 | 3TG4A3-ChAT.tif | FUS | sALS | 3TG4A3 | A3 | A | 3TG4 | FUS |
| **3TG** | 3TG4 | 3TG4A4-ChAT.tif | FUS | sALS | 3TG4A4 | A4 | A | 3TG4 | FUS |
| **3TG** | 3TG4 | 3TG4A5-ChAT.tif | FUS | sALS | 3TG4A5 | A5 | A | 3TG4 | FUS |
| **3TG** | 3TG4 | 3TG4A6-ChAT.tif | FUS | sALS | 3TG4A6 | A6 | A | 3TG4 | FUS |
| **3TG** | 3TG4 | 3TG4A7-ChAT.tif | FUS | sALS | 3TG4A7 | A7 | A | 3TG4 | FUS |
| **3TG** | 3TG4 | 3TG4A8-ChAT.tif | FUS | sALS | 3TG4A8 | A8 | A | 3TG4 | FUS |
| **3TG** | 3TG5 | 3TG5A1 | FUS | sALS | 3TG5A1 | A1 | A | 3TG5 | FUS |
| **3TG** | 3TG5 | 3TG5A2 | FUS | sALS | 3TG5A2 | A2 | A | 3TG5 | FUS |
| **3TG** | 3TG5 | 3TG5A3 | FUS | sALS | 3TG5A3 | A3 | A | 3TG5 | FUS |
| **3TG** | 3TG5 | 3TG5A4 | FUS | sALS | 3TG5A4 | A4 | A | 3TG5 | FUS |
| **3TG** | 3TG5 | 3TG5A5 | FUS | sALS | 3TG5A5 | A5 | A | 3TG5 | FUS |
| **3TG** | 3TG5 | 3TG5A6 | FUS | sALS | 3TG5A6 | A6 | A | 3TG5 | FUS |
| **3TG** | 3TG6 | 3TG6A1 | FUS | sALS | 3TG6A1 | A1 | A | 3TG6 | FUS |
| **3TG** | 3TG6 | 3TG6A2 | FUS | sALS | 3TG6A2 | A2 | A | 3TG6 | FUS |
| **3TG** | 3TG6 | 3TG6A3 | FUS | sALS | 3TG6A3 | A3 | A | 3TG6 | FUS |
| **3TG** | 3TG6 | 3TG6A4 | FUS | sALS | 3TG6A4 | A4 | A | 3TG6 | FUS |
| **3TG** | 3TG6 | 3TG6A5 | FUS | sALS | 3TG6A5 | A5 | A | 3TG6 | FUS |
| **3TG** | 3TG6 | 3TG6A6 | FUS | sALS | 3TG6A6 | A6 | A | 3TG6 | FUS |
| **3TG** | 3TG6 | 3TG6A7 | FUS | sALS | 3TG6A7 | A7 | A | 3TG6 | FUS |
| **3TG** | 3TG6 | 3TG6A8 | FUS | sALS | 3TG6A8 | A8 | A | 3TG6 | FUS |
| **4KI** | 4Ki1 | 4KI1B1L-ChAT.tif | FUS | CTRL | 4Ki1B1L | B1L | BL | 4Ki1 | FUS |
| **4KI** | 4Ki1 | 4KI1B1M-ChAT.tif | FUS | CTRL | 4Ki1B1M | B1M | BM | 4Ki1 | FUS |
| **4KI** | 4Ki1 | 4KI1B1R-ChAT.tif | FUS | CTRL | 4Ki1B1R | B1R | BR | 4Ki1 | FUS |
| **4KI** | 4Ki1 | 4KI1B2L-ChAT.tif | FUS | CTRL | 4Ki1B2L | B2L | BL | 4Ki1 | FUS |
| **4KI** | 4Ki1 | 4KI1B2M-ChAT.tif | FUS | CTRL | 4Ki1B2M | B2M | BM | 4Ki1 | FUS |
| **4KI** | 4Ki1 | 4KI1B2R-ChAT.tif | FUS | CTRL | 4Ki1B2R | B2R | BR | 4Ki1 | FUS |
| **4KI** | 4Ki1 | 4KI1B3L-ChAT.tif | FUS | CTRL | 4Ki1B3L | B3L | BL | 4Ki1 | FUS |
| **4KI** | 4Ki1 | 4KI1B3M-ChAT.tif | FUS | CTRL | 4Ki1B3M | B3M | BM | 4Ki1 | FUS |
| **4KI** | 4Ki1 | 4KI1B3R-ChAT.tif | FUS | CTRL | 4Ki1B3R | B3R | BR | 4Ki1 | FUS |
| **4KI** | 4Ki1 | 4KI1B4L-ChAT.tif | FUS | CTRL | 4Ki1B4L | B4L | BL | 4Ki1 | FUS |
| **4KI** | 4Ki1 | 4KI1B4R-ChAT.tif | FUS | CTRL | 4Ki1B4R | B4R | BR | 4Ki1 | FUS |
| **4KI** | 4Ki1 | 4KI1B5R-ChAT.tif | FUS | CTRL | 4Ki1B5R | B5R | BR | 4Ki1 | FUS |
| **4KI** | 4Ki1 | 4KI1B6R | FUS | CTRL | 4Ki1B6R | B6R | BR | 4Ki1 | FUS |
| **4KI** | 4Ki2 | 4KI2B1R-ChAT.tif | FUS | CTRL | 4Ki2B1R | B1R | BR | 4Ki2 | FUS |
| **4KI** | 4Ki2 | 4KI2B2M-ChAT.tif | FUS | CTRL | 4Ki2B2M | B2M | BM | 4Ki2 | FUS |
| **4KI** | 4Ki2 | 4KI2B2R-ChAT.tif | FUS | CTRL | 4Ki2B2R | B2R | BR | 4Ki2 | FUS |
| **4KI** | 4Ki2 | 4KI2B3M-ChAT.tif | FUS | CTRL | 4Ki2B3M | B3M | BM | 4Ki2 | FUS |
| **4KI** | 4Ki2 | 4KI2B4R-ChAT.tif | FUS | CTRL | 4Ki2B4R | B4R | BR | 4Ki2 | FUS |
| **4KI** | 4Ki2 | 4KI2B1m | FUS | CTRL | 4Ki2B1m | B1m | Bm | 4Ki2 | FUS |
| **4KI** | 4Ki2 | 4KI2B3R | FUS | CTRL | 4Ki2B3R | B3R | BR | 4Ki2 | FUS |
| **4KI** | 4Ki3 | 4KI3B1L-ChAT.tif | FUS | CTRL | 4Ki3B1L | B1L | BL | 4Ki3 | FUS |
| **4KI** | 4Ki3 | 4KI3B1M-ChAT.tif | FUS | CTRL | 4Ki3B1M | B1M | BM | 4Ki3 | FUS |
| **4KI** | 4Ki3 | 4KI3B1R-ChAT.tif | FUS | CTRL | 4Ki3B1R | B1R | BR | 4Ki3 | FUS |
| **4KI** | 4Ki3 | 4KI3B2L-ChAT.tif | FUS | CTRL | 4Ki3B2L | B2L | BL | 4Ki3 | FUS |
| **4KI** | 4Ki3 | 4KI3B2M-ChAT.tif | FUS | CTRL | 4Ki3B2M | B2M | BM | 4Ki3 | FUS |
| **4KI** | 4Ki3 | 4KI3B2R-ChAT.tif | FUS | CTRL | 4Ki3B2R | B2R | BR | 4Ki3 | FUS |
| **4KI** | 4Ki3 | 4KI3B3L-ChAT.tif | FUS | CTRL | 4Ki3B3L | B3L | BL | 4Ki3 | FUS |
| **4KI** | 4Ki3 | 4KI3B3M-ChAT.tif | FUS | CTRL | 4Ki3B3M | B3M | BM | 4Ki3 | FUS |
| **4KI** | 4Ki3 | 4KI3B3R-ChAT.tif | FUS | CTRL | 4Ki3B3R | B3R | BR | 4Ki3 | FUS |
| **4KI** | 4Ki3 | 4KI3B4M-ChAT.tif | FUS | CTRL | 4Ki3B4M | B4M | BM | 4Ki3 | FUS |
| **4KI** | 4Ki3 | 4KI3B4R | FUS | CTRL | 4Ki3B4R | B4R | BR | 4Ki3 | FUS |
| **4KI** | 4Ki3 | 4KI3B5R | FUS | CTRL | 4Ki3B5R | B5R | BR | 4Ki3 | FUS |
| **4KI** | 4Ki4 | 4KI4B1M-ChAT.tif | FUS | sALS | 4Ki4B1M | B1M | BM | 4Ki4 | FUS |
| **4KI** | 4Ki4 | 4KI4B1R-ChAT.tif | FUS | sALS | 4Ki4B1R | B1R | BR | 4Ki4 | FUS |
| **4KI** | 4Ki4 | 4KI4B2R-ChAT.tif | FUS | sALS | 4Ki4B2R | B2R | BR | 4Ki4 | FUS |
| **4KI** | 4Ki4 | 4KI4B3R-ChAT.tif | FUS | sALS | 4Ki4B3R | B3R | BR | 4Ki4 | FUS |
| **4KI** | 4Ki4 | 4KI4B4R-ChAT.tif | FUS | sALS | 4Ki4B4R | B4R | BR | 4Ki4 | FUS |
| **4KI** | 4Ki4 | 4KI4BM1 | FUS | sALS | 4Ki4BM1 | BM1 | BM | 4Ki4 | FUS |
| **4KI** | 4Ki4 | 4KI4BM2 | FUS | sALS | 4Ki4BM2 | BM2 | BM | 4Ki4 | FUS |
| **4KI** | 4Ki5 | 4KI5B1L-ChAT.tif | FUS | sALS | 4Ki5B1L | B1L | BL | 4Ki5 | FUS |
| **4KI** | 4Ki5 | 4KI5B1M-ChAT.tif | FUS | sALS | 4Ki5B1M | B1M | BM | 4Ki5 | FUS |
| **4KI** | 4Ki5 | 4KI5B1R-ChAT.tif | FUS | sALS | 4Ki5B1R | B1R | BR | 4Ki5 | FUS |
| **4KI** | 4Ki5 | 4KI5B2M-ChAT.tif | FUS | sALS | 4Ki5B2M | B2M | BM | 4Ki5 | FUS |
| **4KI** | 4Ki5 | 4KI5B2R-ChAT.tif | FUS | sALS | 4Ki5B2R | B2R | BR | 4Ki5 | FUS |
| **4KI** | 4Ki6 | 4KI6B2R-ChAT.tif | FUS | sALS | 4Ki6B2R | B2R | BR | 4Ki6 | FUS |
| **4KI** | 4Ki6 | 4KI6B3R-ChAT.tif | FUS | sALS | 4Ki6B3R | B3R | BR | 4Ki6 | FUS |
| **4KI** | 4Ki6 | 4KI6B4R-ChAT.tif | FUS | sALS | 4Ki6B4R | B4R | BR | 4Ki6 | FUS |
| **4KI** | 4Ki6 | 4KI6BL1lsm-ChAT.tif | FUS | sALS | 4Ki6BL1lsm | BL1lsm | BLlsm | 4Ki6 | FUS |
| **4KI** | 4Ki6 | 4KI6BL2-ChAT.tif | FUS | sALS | 4Ki6BL2 | BL2 | BL | 4Ki6 | FUS |
| **4KI** | 4Ki6 | 4KI6BL3-ChAT.tif | FUS | sALS | 4Ki6BL3 | BL3 | BL | 4Ki6 | FUS |
| **4KI** | 4Ki6 | 4KI6BL4-ChAT.tif | FUS | sALS | 4Ki6BL4 | BL4 | BL | 4Ki6 | FUS |
| **4KI** | 4Ki6 | 4KI6BM2-ChAT.tif | FUS | sALS | 4Ki6BM2 | BM2 | BM | 4Ki6 | FUS |
| **4KI** | 4Ki6 | 4KI6BM3-ChAT.tif | FUS | sALS | 4Ki6BM3 | BM3 | BM | 4Ki6 | FUS |
| **4KI** | 4Ki6 | 4KI6BM4-ChAT.tif | FUS | sALS | 4Ki6BM4 | BM4 | BM | 4Ki6 | FUS |
| **4KI** | 4Ki6 | 4KI6BM5-ChAT.tif | FUS | sALS | 4Ki6BM5 | BM5 | BM | 4Ki6 | FUS |
| **4KI** | 4Ki6 | 4KI6B1R | FUS | sALS | 4Ki6B1R | B1R | BR | 4Ki6 | FUS |
| **4KI** | 4Ki6 | 4KI6BM1 | FUS | sALS | 4Ki6BM1 | BM1 | BM | 4Ki6 | FUS |
| **4KI** | 4Ki1 | 2ki1Dc1-ChAT.tif | SFPQ | CTRL | 4Ki1Dc1 | Dc1 | Dc | 4Ki1 | SFPQ |
| **4KI** | 4Ki1 | 2ki1Dc3-ChAT.tif | SFPQ | CTRL | 4Ki1Dc3 | Dc3 | Dc | 4Ki1 | SFPQ |
| **4KI** | 4Ki1 | 2ki1Dl1-ChAT.tif | SFPQ | CTRL | 4Ki1Dl1 | Dl1 | Dl | 4Ki1 | SFPQ |
| **4KI** | 4Ki1 | 2ki1Dr1-ChAT.tif | SFPQ | CTRL | 4Ki1Dr1 | Dr1 | Dr | 4Ki1 | SFPQ |
| **4KI** | 4Ki1 | 2ki1Dr2-ChAT.tif | SFPQ | CTRL | 4Ki1Dr2 | Dr2 | Dr | 4Ki1 | SFPQ |
| **4KI** | 4Ki1 | 2ki1Dc2 | SFPQ | CTRL | 4Ki1Dc2 | Dc2 | Dc | 4Ki1 | SFPQ |
| **4KI** | 4Ki2 | 2ki2Dc1-ChAT.tif | SFPQ | CTRL | 4Ki2Dc1 | Dc1 | Dc | 4Ki2 | SFPQ |
| **4KI** | 4Ki2 | 2ki2Dc3-ChAT.tif | SFPQ | CTRL | 4Ki2Dc3 | Dc3 | Dc | 4Ki2 | SFPQ |
| **4KI** | 4Ki2 | 2ki2Dr2-ChAT.tif | SFPQ | CTRL | 4Ki2Dr2 | Dr2 | Dr | 4Ki2 | SFPQ |
| **4KI** | 4Ki2 | 2ki2Dr3-ChAT.tif | SFPQ | CTRL | 4Ki2Dr3 | Dr3 | Dr | 4Ki2 | SFPQ |
| **4KI** | 4Ki2 | 2ki2Dc2 | SFPQ | CTRL | 4Ki2Dc2 | Dc2 | Dc | 4Ki2 | SFPQ |
| **4KI** | 4Ki3 | 2ki3DC1-ChAT.tif | SFPQ | CTRL | 4Ki3DC1 | DC1 | DC | 4Ki3 | SFPQ |
| **4KI** | 4Ki3 | 2ki3Dc3-ChAT.tif | SFPQ | CTRL | 4Ki3Dc3 | Dc3 | Dc | 4Ki3 | SFPQ |
| **4KI** | 4Ki3 | 2ki3Dc4-ChAT.tif | SFPQ | CTRL | 4Ki3Dc4 | Dc4 | Dc | 4Ki3 | SFPQ |
| **4KI** | 4Ki3 | 2ki3Dc5-ChAT.tif | SFPQ | CTRL | 4Ki3Dc5 | Dc5 | Dc | 4Ki3 | SFPQ |
| **4KI** | 4Ki3 | 2ki3Dl1-ChAT.tif | SFPQ | CTRL | 4Ki3Dl1 | Dl1 | Dl | 4Ki3 | SFPQ |
| **4KI** | 4Ki3 | 2ki3Dl2-ChAT.tif | SFPQ | CTRL | 4Ki3Dl2 | Dl2 | Dl | 4Ki3 | SFPQ |
| **4KI** | 4Ki3 | 2ki3Dl3-ChAT.tif | SFPQ | CTRL | 4Ki3Dl3 | Dl3 | Dl | 4Ki3 | SFPQ |
| **4KI** | 4Ki3 | 2ki3Dr1-ChAT.tif | SFPQ | CTRL | 4Ki3Dr1 | Dr1 | Dr | 4Ki3 | SFPQ |
| **4KI** | 4Ki3 | 2ki3Dr2-ChAT.tif | SFPQ | CTRL | 4Ki3Dr2 | Dr2 | Dr | 4Ki3 | SFPQ |
| **4KI** | 4Ki3 | 2ki3Dc2 | SFPQ | CTRL | 4Ki3Dc2 | Dc2 | Dc | 4Ki3 | SFPQ |
| **4KI** | 4Ki4 | 2ki4Dc1-ChAT.tif | SFPQ | sALS | 4Ki4Dc1 | Dc1 | Dc | 4Ki4 | SFPQ |
| **4KI** | 4Ki4 | 2ki4Dc2-ChAT.tif | SFPQ | sALS | 4Ki4Dc2 | Dc2 | Dc | 4Ki4 | SFPQ |
| **4KI** | 4Ki4 | 2ki4Dc3-ChAT.tif | SFPQ | sALS | 4Ki4Dc3 | Dc3 | Dc | 4Ki4 | SFPQ |
| **4KI** | 4Ki4 | 2ki4Dl1-ChAT.tif | SFPQ | sALS | 4Ki4Dl1 | Dl1 | Dl | 4Ki4 | SFPQ |
| **4KI** | 4Ki4 | 2ki4Dl2-ChAT.tif | SFPQ | sALS | 4Ki4Dl2 | Dl2 | Dl | 4Ki4 | SFPQ |
| **4KI** | 4Ki4 | 2ki4Dr1-ChAT.tif | SFPQ | sALS | 4Ki4Dr1 | Dr1 | Dr | 4Ki4 | SFPQ |
| **4KI** | 4Ki4 | 2ki4Dr3-ChAT.tif | SFPQ | sALS | 4Ki4Dr3 | Dr3 | Dr | 4Ki4 | SFPQ |
| **4KI** | 4Ki4 | 2ki4Dr4-ChAT.tif | SFPQ | sALS | 4Ki4Dr4 | Dr4 | Dr | 4Ki4 | SFPQ |
| **4KI** | 4Ki5 | 2ki5Dc3-ChAT.tif | SFPQ | sALS | 4Ki5Dc3 | Dc3 | Dc | 4Ki5 | SFPQ |
| **4KI** | 4Ki5 | 2ki5Dl1-ChAT.tif | SFPQ | sALS | 4Ki5Dl1 | Dl1 | Dl | 4Ki5 | SFPQ |
| **4KI** | 4Ki5 | 2ki5Dr1-ChAT.tif | SFPQ | sALS | 4Ki5Dr1 | Dr1 | Dr | 4Ki5 | SFPQ |
| **4KI** | 4Ki5 | 2ki5Dr2-ChAT.tif | SFPQ | sALS | 4Ki5Dr2 | Dr2 | Dr | 4Ki5 | SFPQ |
| **4KI** | 4Ki5 | 2ki5Dr3-ChAT.tif | SFPQ | sALS | 4Ki5Dr3 | Dr3 | Dr | 4Ki5 | SFPQ |
| **4KI** | 4Ki5 | 2ki5Dc1 | SFPQ | sALS | 4Ki5Dc1 | Dc1 | Dc | 4Ki5 | SFPQ |
| **4KI** | 4Ki5 | 2ki5Dc2 | SFPQ | sALS | 4Ki5Dc2 | Dc2 | Dc | 4Ki5 | SFPQ |
| **4KI** | 4Ki6 | 2ki6DR2-ChAT.tif | SFPQ | sALS | 4Ki6DR2 | DR2 | DR | 4Ki6 | SFPQ |
| **4KI** | 4Ki6 | 2ki6DR3-ChAT.tif | SFPQ | sALS | 4Ki6DR3 | DR3 | DR | 4Ki6 | SFPQ |
| **4KI** | 4Ki6 | 2ki6DR4-ChAT.tif | SFPQ | sALS | 4Ki6DR4 | DR4 | DR | 4Ki6 | SFPQ |
| **4KI** | 4Ki6 | 2ki6DR5-ChAT.tif | SFPQ | sALS | 4Ki6DR5 | DR5 | DR | 4Ki6 | SFPQ |
| **4KI** | 4Ki6 | 2ki6DR1 | SFPQ | sALS | 4Ki6DR1 | DR1 | DR | 4Ki6 | SFPQ |

**Table S2 |** List of images used for FUS and SFPQ cellular localisation in [(Luisier *et al.*, 2018; Tyzack *et al.*, 2019)](https://paperpile.com/c/dbswo3/0HdLy+vHVXw); human data.


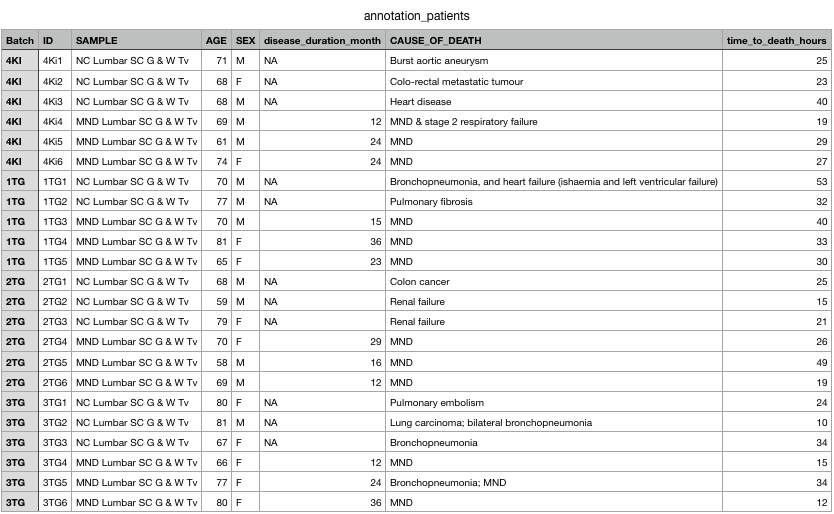


**Table S3 |** Description of the donors from which PMTs were obtained.

asasdaasasdassedical Signal Proce

**MATERIALS AND METHODS**

**Compliance with ethical standards**

Experimental protocols were all carried out according to approved regulations and guidelines by the UCLH’s National Hospital for Neurology and Neurosurgery and UCL Queen Square Institute of Neurology joint research ethics committee (09/0272). The human post-mortem spinal cord samples were obtained from the tissue bank NeuroResource, UCL Queen Square Institute of Neurology, London, UK. Samples were donated to the tissue bank with written tissue donor informed consent following ethical review by the NHS NRES Committee London–Central and stored under a Research Sector Licence from the UK Human Tissue Authority (HTA).

### **Animals, transgenic models and tissue processing**

All experiments were carried out following the guidelines of the UCL Institute of Neurology Genetic Manipulation and Ethic Committees and in accordance with the European Community Council Directive of November 24, 1986 (86/609/EEC). Animal experiments were undertaken under a Licence from the UK Home Office in accordance with the Animals (Scientific Procedures) Act 1986 (Amended Regulations 2012) and were approved by the Ethical Review Panel of the Institute of Neurology. The human spinal cord samples were obtained from the tissue bank NeuroResource, UCL Institute of Neurology, London, UK. Samples were donated to the tissue bank with written tissue donor informed consent following ethical review by the NHS NRES Committee London–Central and stored under a Research Sector Licence from the UK Human Tissue Authority (HTA). Animals, transgenic models and tissue processing were performed as in [(Luisier *et al.*, 2018; Tyzack *et al.*, 2019)](https://paperpile.com/c/dbswo3/vHVXw+0HdLy). Indeed these data are utilised in the current manuscript and no additional animal usage was required.

###

###

### **Automated image processing**

Both mouse tissues and patient post-mortem samples were sectioned and immunostaining as described in our previous studies [(Luisier *et al.*, 2018; Tyzack *et al.*, 2019)](https://paperpile.com/c/dbswo3/vHVXw+0HdLy). Raw images were processed by combining the following open source softwares (and packages) for automatic segmentation and measurements of cell features: ImageJ, [(Schneider *et al.*, 2012)](https://paperpile.com/c/dbswo3/orcJn), Ilastik [(Berg *et al.*, 2019*a*)](https://paperpile.com/c/dbswo3/DRfYk) and Cellprofiler [(Carpenter *et al.*, 2006)](https://paperpile.com/c/dbswo3/d9g8y) Diagrams of the mouse and human pipelines are shown in **Supplementary Fig.1**.

**Mouse Tissue pipeline (Supplementary Fig. 1A)**

*Preprocessing:* choline acetyltransferase (ChAT) immunolabeling is a reliable MN marker here used for MN segmentation. To facilitate the segmentation, we first enhanced the contrast, subtracted the background and applied gaussian and median filters to each individual image, using the base scripts provided within the main FiJi (ImageJ) release [(Schneider *et al.*, 2012)](https://paperpile.com/c/dbswo3/orcJn). Additionally we used the particle detection and image calculator packages in ImageJ to remove objects smaller than 2000px, as these were considered staining artifacts. A custom script combining these preprocessing steps in ImageJ is accessible in [Zenodo](http://doi.org/10.5281/zenodo.3985099) under the accession number 3985099. Notably the modified images were only used for segmentation while original images were used for cell profiling.

*Pixel classification:* a randomly selected subset of the preprocessed images (ranging from 8 to 15) was used for training using a parallel random forest (VIGRA) algorithm in Ilastik (see labels and training set examples provided in Zenodo) for automated MNs segmentation. For nuclei segmentation, a subset of DAPI and ChAT fluorescent images were next combined and training was undertaken in Ilastik (see labels and training set examples provided in Zenodo).

*Automated image analysis:* the three binary masks generated by the pixel classification algorithm (MNs, nuclei, inclusions) were added to the original channels and then used as a dataset for automated analysis in Cellprofiler (see pipeline provided in Zenodo). Here the masks were converted to objects for further processing. The inclusion-objects were subtracted from MN-objects and nuclei-objects to exclude measurements of staining artifacts. Then the resulting ‘clean’ nuclei-objects were used to detect MNs including nuclei. This is a crucial step for processing sliced images where nuclei can be physically separated from their corresponding MN. We continued processing these selected MNs by subtracting the nuclei-object and therefore defining the cytoplasm of this cell. As a final step, for each MN, texture, intensity, intensity distribution, size and shape-related measurements were acquired in MN-, nuclei, cytoplasm-objects using the original (non preprocessed) fluorescent images of DAPI, FUS, SFPQ and ChAT.

**Post-mortem tissue** (**Supplementary Fig. 1B**)

*Preprocessing:* to facilitate MN segmentation, we first enhanced the contrast and equalized the histogram of the fluorescent channels (DAPI, ChAT, FUS) using existing packages within FiJi (ImageJ - see preprocessing scripts provided in Zenodo), which served to counteract the varying background noise of the images. These preprocessing steps have been adopted to facilitate object detection, and the modified images were not used in the following automated analysis steps that directly measure staining intensity or densitometry.

*Pixel Classification:* A randomly selected representative subset of the preprocessed images (26 in total) was then used for training a parallel random forest (VIGRA) algorithm in Ilastik (see labels and training set examples provided in Zenodo) to identify MNs from background, and to exclude the corpora amylacea (age-related insoluble accumulations detectable in postmortem tissue that tend to present non-specific immunolabeling across all channels, and need to therefore be identified and excluded from the analysis, henceforth identified as “inclusions”). The training was performed on preprocessed images using 3-channels (DAPI, ChAT, FUS).

*Automated Image Analysis:* The binary masks generated by the pixel classification (ML-MNs) were then added to the original channels and used as a dataset for automated analysis in Cellprofiler (see pipeline provided in Zenodo). In Cellprofiler we implemented steps to detect nuclei as a secondary structure based on the MNs identified in Ilastik (ML-MNs) overlaid to the DAPI channels, and the cytoplasm was determined by subtraction of DAPI to the identified MNs as a tertiary structure. The resulting nuclei- and cytoplasm-objects were used to measure the texture, intensity, intensity distribution, size, shape, colocalization and radial distribution for each MN using the (non preprocessed) fluorescent images of DAPI, FUS, SFPQ and ChAT.

**Preprocessing of high-content imaging data**

Measurements from whole MNs, nuclear and cytoplasmic compartments were merged to form a single data matrix. Subsequent analyses were performed with the R statistical package version 3.3.1, Bioconductor libraries version 3.3 (R Core Team. R: A Language and Environment for Statistical Computing. Vienna, Austria: R Foundation for Statistical Computing; 2013), and Python 3.5.3 in a single Jupyter notebook framework. MNs with cytoplasmic compartment areas smaller than nuclear compartment areas were removed for primary analysis in order to ensure similar positioning of the cells. As normal distributions make it easier to work with numeric values from a mathematical, statistical, and computational point of view [(Caicedo *et al.*, 2017)](https://paperpile.com/c/dbswo3/NgnhK), we iteratively tested the normality of the measurements across the cells and log-transformed to obtain approximate normal distributions for features that have highly skewed values or require range correction. Additionally we standardized the measurements to ensure approximately normally distributed, mean centered and have standard-deviation.

**Unsupervised characterisation of high-content microscopy data**

Hierarchical clustering of the cells based on their morphological profiles has been carried out using weighted-average linkage applied to euclidean distances. Singular Value Decomposition (SVD) has been performed on the hundreds of measurements across hundreds of cells. Selection of the components maximally capturing variance in gene expression resulted in a subset of components to focus on, which can be interpreted as characteristic cell phenotype. Linear mixed model (LMM) was then used to test the association between each of the $n$ first selected principal components and vcpALS phenotype (VCP-mutant MNs), comALS phenotype (VCP- and SOD1-mutant MNs), or sALS phenotype, accounting for idiosyncratic variations due to the animals or individuals where the cells originated. The right singular vectors were used to generate the PCA scatter plots of the MNs projected on the principal components, while the left singular vectors were used to extract the contribution of each measurement to each component.

**Automated identification of MNs subpopulation**

Gaussian Mixture Models (GMM) are a powerful label-free probabilistic distribution-based clustering algorithm that have been shown to be effective in capturing subpopulations in imaging data [(Slack *et al.*, 2008, Loo *et al.*, 2009*b*)](https://paperpile.com/c/dbswo3/2UZsi+m7jtV). In this model, the subpopulations and their proportions correspond to mixture centers and mixture prior probabilities, respectively. Both of these quantities are considered as unknown parameters and were estimated using the expectation maximization (EM) algorithm. The algorithm was initialized with unit covariance and the centroid positions obtained using the k-means algorithm. The starting positions of the centroids in the k-means algorithm were initialized randomly, meaning the algorithm is nondeterministic. Notably label-independent classifiers such as GMM do not require prior knowledge regarding the disease status of the cells and therefore the distinction between healthy and sick cells is not a prerequisite for this method.

To compare against the label-independent GMM-based classifier, we also selected two label-dependent probabilistic classifiers, namely Logistic Regression (LR) and a multilayer perceptron (MLP) neural network with one hidden layer. LR and MLP can deal with more complex patterns in data whilst relying on labeling of the cells. In particular MLPs decision boundaries can be nonlinear. In LR the contribution of parameters (coefficients and intercept) can be easily interpreted, which is not always the case with the parameters of a neural network. In these two cases, the a priori distinction between healthy and sick cells was made according to the tissue origin, for example the MNs were labeled as sick when they originated from SOD1- or FUS-mutant mice, or from sALS patients, and healthy otherwise. Prior to training, we splitted the data into train (70% of the cells) and test sets (30% of the cells) in stratified fashion by conserving the relative fraction of cells originating from each animal or individual. The regularization strength of the LR has been optimised using 10-fold cross-validation over a parameter grid and selecting against the best accuracy score. The hyperparameters of the MLP, namely the regularization term, the hidden layer size, the solver and the activation were selected using 5-fold cross-validation over a parameter space and selecting against the best accuracy score. GMM, LR and MLP have been trained and optimised in the SciPy environment using the scikit-learn library [(Pedregosa *et al.*, 2011)](https://paperpile.com/c/dbswo3/15CEk). The performances of each classifier were then compared using the Receiver Operational Characteristics (ROC) curves and the Area under the curves (AUC).

**Relative contribution of cell measurements**

The relative contributions of the individual measurements to the different classifiers were obtained as follows. For GMM-based classifiers, the left singular vectors of the components used for GMM modeling were extracted and then converted in relative quantities by dividing each vector by its sum. For LR classifiers, the weights were extracted and similarly converted into relative quantities by dividing each weight by the sum of all the weights for a given LR. MLP uses equation (1) to model the probability of each MNs to be sick given the observed measurement vector X of dimension $[1\times m]$ where *m* is the number of measurements, $w_{i}$ is the weight of the *i*th perceptron of the hidden layer, $v_{ij}$ is the weight of the *j*th cell measurement in the *i*th hidden perceptron, and $n$is the number of perceptrons in the hidden layer.

$P\left( sick | X \right)=\sum_{i=1}^{n} w_{i} tanh\left( \sum_{j=1}^{m} v_{ij}\times x_{j}+v_{oi} \right)$ (1)

To calculate the relative contribution $\hat{r_{j}}$ of each measurement *j* to the MLP classifier, we first extracted the global relative contribution using equation (2).

$r{}_{j}=\sum_{i=1}^{n} w_{i}\times v_{ij}$ (2)

In order to get then the relative contribution to the classifier, we finally divided all contributions by the sum of all contributors as shown in equation (3).

$\hat{r_{j}}=\frac{r_{j}}{\sum_{i=1}^{m} {rj}}$ (3)

**Scoring metrics**

GMM, LR and MLP are probabilistic classifiers which output a per-cell posterior probability *P* to be sick given the observed phenotype. The biological interpretation of this probability relates to the confidence for a given cell to be either sick or healthy given the model. We also transformed these disease probabilities using equation (4) to generate alternative scoring metrics that relate to the ‘disease severity’ expected to better reflect on the possibility for two cells to exhibit very different degrees of aberrant phenotypes while having similarly high probability to be sick (**Supplementary Fig. 3**).

$S=-log\left( 1-P \right)$ (4)

The per-cell disease profiles were finally constructed using 1) cell-level probability of the disease and 2) cell-level disease severity score. These vectors are expected to capture the disease status of each cell as captured by the cellular morphology. From these two metrics we also extracted 1) per-animal probability to be sick by averaging the probabilities across all cells for each animal, 2) per-animal disease severity by averaging the severity scores across all cells for each animal, 3) per-animal fraction of sick cells by computing the fraction of cells with probability above 0.0 to be sick in each animal.
