# Supplementary material for: Automated and unbiased discrimination of ALS from control tissue at single cell resolution
Source: Brain Pathol. 2021 Feb 11;31(4):e12937. doi: 10.1111/bpa.12937 (PMC8412073; doi:10.1111/bpa.12937)
Supplement: Supplementary file 2 — Supplementary Material FIGURE S1 (A) Diagram representing the image processing workflow for mouse tissue immunolabeled for FUS, SFPQ, ChAT and counterstained with DAPI. To facilitate the MN segmentation, we first applied contrast enhancement, background correction and application of gaussian blur and median filters to ChAT stained images using ImageJ (35). Next we used a subset of these preprocessed ChAT images to train a pixel classification algorithm in Ilastik (11) for automated identification of artifacts and MNs. In parallel, automated nuclear segmentation was trained in Ilastik on randomly selected subsets of overlaid ChAT and DAPI images. Finally, all generated segmentations were added to the original dataset as additional channels, and used to identify the cytoplasm of MNs and perform automated densitometry and morphometric measurements in each compartment (cytoplasm, nucleus, whole MNs) in CellProfiler (14). (B) Diagram representing the image processing workflow for human post‐mortem tissue immunolabeled for ChAT, FUS or SFPQ, and counterstained with DAPI. Individual channels were preprocessed in ImageJ for contrast enhancement and histogram equalisation. Next automated MN segmentation was trained using a subset of the original images using IIastik based on the three channels (SFPQ, DAPI, ChAT). Next DAPI channel and the masked MNs were provided to CellProfiler for automated segmentation of nuclei and cytoplasm followed by automatic acquisition of single‐cell measurements in each compartment FIGURE S2 Fraction of explained variance captured by the first 31 principal components that captures 90% of the signal. Shannon Entropy of 0.65 indicates that the information in the data is well distributed among the principal components FIGURE S3 Comparison between disease probability P and disease severity S scores showing how similarly high disease probability can exhibit large differences in disease severity FIGURE S4 (A, B) MNs predicted probability distribution (l [file BPA-31-e12937-s001.pdf]

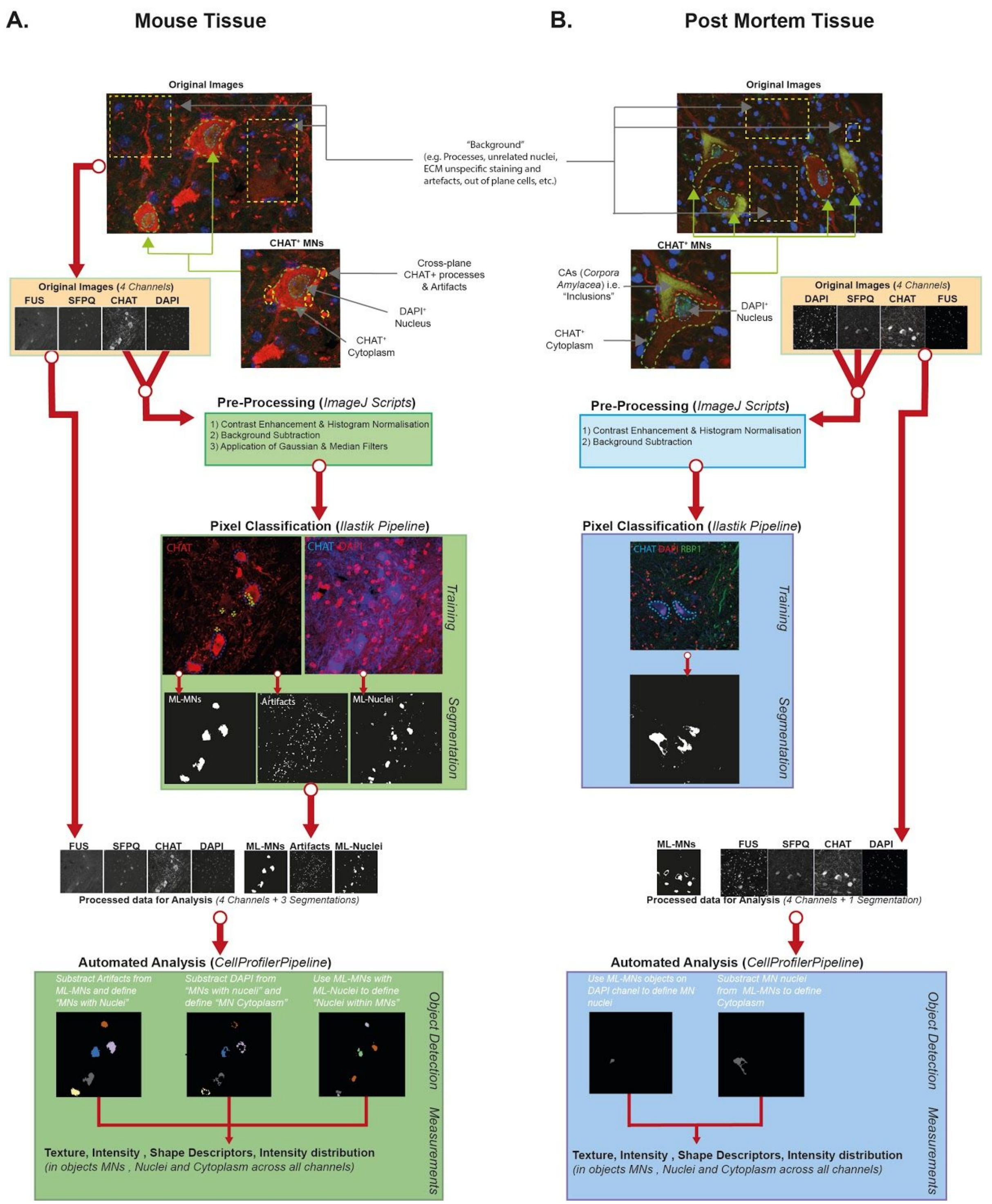

Supplementary Figure 1 | (**A**) Diagram representing the image processing workflow for mouse tissue immunolabeled for FUS, SFPQ, ChAT and counterstained with DAPI. To

facilitate the MN segmentation, we first applied contrast enhancement, background correction and application of gaussian blur and median filters to ChAT stained images using ImageJ (Schneider *et al.*, 2012). Next we used a subset of these preprocessed ChAT images to train a pixel classification algorithm in Ilastik (Berg *et al.*, 2019*a*) for automated identification of artifacts and MNs. In parallel, automated nuclear segmentation was trained in Ilastik on randomly selected subsets of overlaid ChAT and DAPI images. Finally, all generated segmentations were added to the original dataset as additional channels, and used to identify the cytoplasm of MNs and perform automated densitometry and morphometric measurements in each compartment (cytoplasm, nucleus, whole MNs) in CellProfiler (Carpenter *et al.*, 2006).(**B**) Diagram representing the image processing workflow for human post-mortem tissue immunolabeled for ChAT, FUS or SFPQ, and counterstained with DAPI. Individual channels were preprocessed in ImageJ for contrast enhancement and histogram equalisation. Next automated MN segmentation was trained using a subset of the original images using Ilastik based on the three channels (SFPQ, DAPI, ChAT). Next DAPI channel and the masked MNs were provided to CellProfiler for automated segmentation of nuclei and cytoplasm followed by automatic acquisition of single-cell measurements in each compartment.

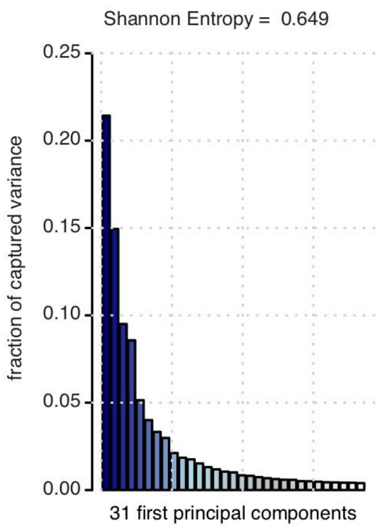

Supplementary Figure 2 | Fraction of explained variance captured by the first 31 principal components that captures 90% of the signal. Shannon Entropy of 0.65 indicates that the information in the data is well distributed among the principal components.

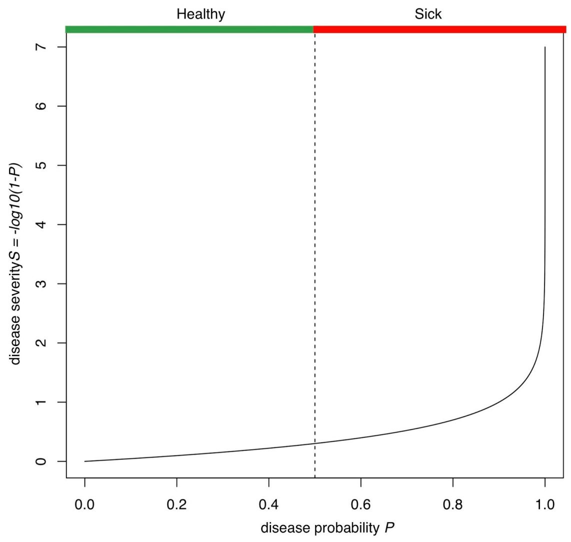

Supplementary Figure 3 | Comparison between disease probability P and disease severity S scores showing how similarly high disease probability can exhibit large differences in disease severity.

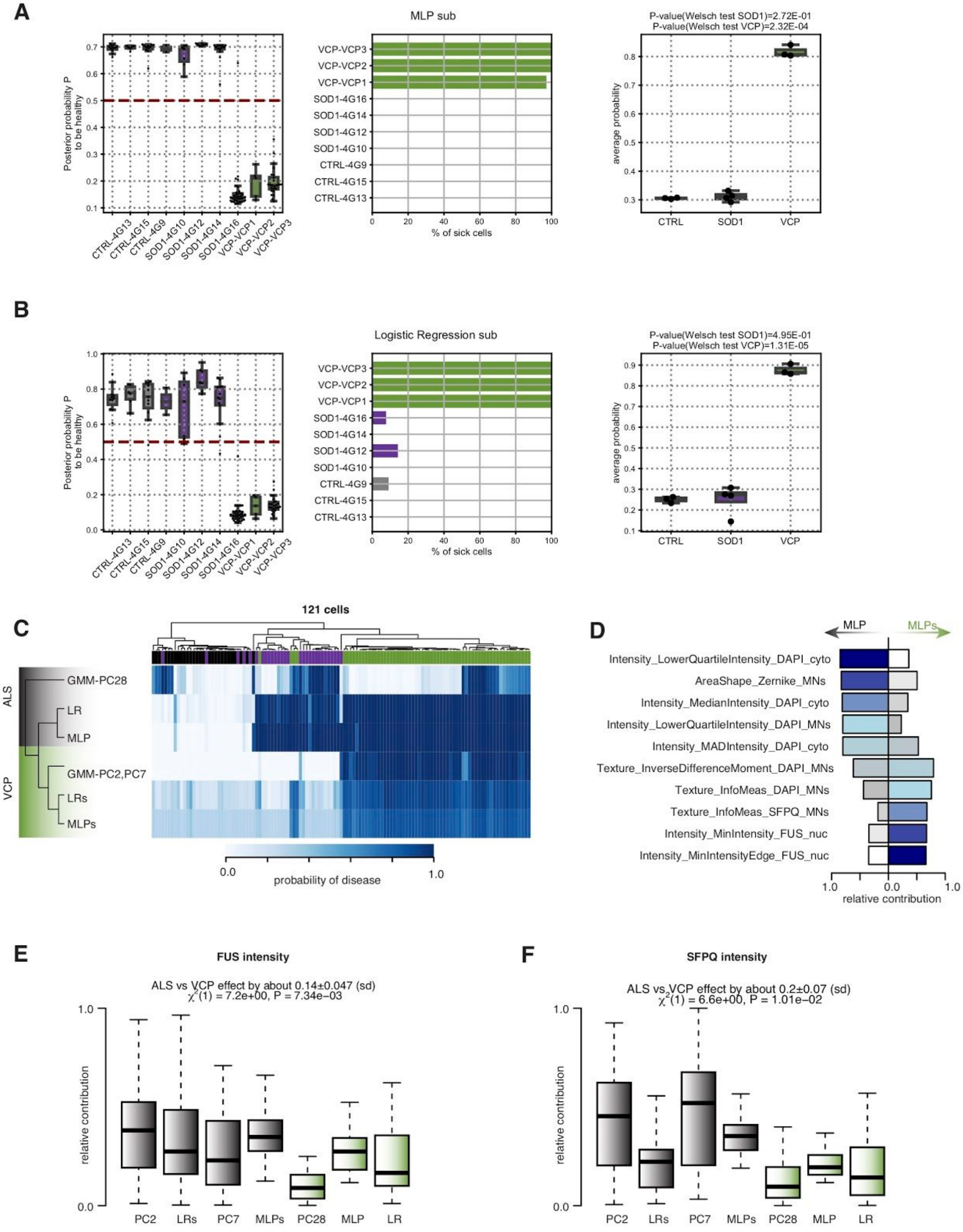
A
MLP sub
P-value(Welsch test SOD1)=2.72E-01
P-value(Welsch test VCP)=2.32E-04
Posterior probability P to be healthy
% of sick cells
average probability
CTRL-4G13
CTRL-4G15
CTRL-4G9
SOD1-4G10
SOD1-4G12
SOD1-4G14
SOD1-4G16
VCP-VCP1
VCP-VCP2
VCP-VCP3
CTRL
SOD1
VCP
B
Logistic Regression sub
P-value(Welsch test SOD1)=4.95E-01
P-value(Welsch test VCP)=1.31E-05
Posterior probability P to be healthy
% of sick cells
average probability
C
121 cells
ALS
VCP
GMM-PC28
LR
MLP
GMM-PC2,PC7
LRs
MLPs
0.0
1.0
probability of disease
D
MLP
MLPs
Intensity_LowerQuartileIntensity_DAPI_cyto
AreaShape_Zernike_MNs
Intensity_MedianIntensity_DAPI_cyto
Intensity_LowerQuartileIntensity_DAPI_MNs
Intensity_MADIntensity_DAPI_cyto
Texture_InverseDifferenceMoment_DAPI_MNs
Texture_InfoMeas_DAPI_MNs
Texture_InfoMeas_SFPQ_MNs
Intensity_MinIntensity_FUS_nuc
Intensity_MinIntensityEdge_FUS_nuc
relative contribution
E
FUS intensity
ALS vs VCP effect by about 0.14±0.047 (sd)
χ2(1) = 7.2e+00, P = 7.34e−03
relative contribution
PC2
LRs
PC7
MLPs
PC28
MLP
LR
F
SFPQ intensity
ALS vs VCP effect by about 0.2±0.07 (sd)
χ2(1) = 6.6e+00, P = 1.01e−02
relative contribution
PC2
LRs
PC7
MLPs
PC28
MLP
LR

Supplementary Figure 4 | (**A**, **B**) MNs predicted probability distribution (*left*), per-animal percentage of sick cells (*centre*), and per-animal disease probability *(right)* as obtained by LR classifier (**A**) and MLP classifier (**B**) trained on data censored for SOD1-mutant cells. (**C**) Heatmap showing the predicted disease probability for the 121 cells. Classifiers are hierarchically clustered using average alog on euclidean distances between disease probability profiles across the 121 cells. Green = *vcpALS* classifiers. Grey = *comALS* classifiers. (**D**) Barplots showing the relative contribution of the top five measurements in MLP and MLPs i.e. *comALS* versus *vcpALS* classifiers. Zernike moments either in the nucleus or the whole MNs contribute largely to ALS but not VCP classifier. Bars are color-coded according to the ranking in contribution for the given classifier, from dark blue to white for high to low ranking. (**E,F**) Boxplots showing the relative contribution of FUS (**E**) and SFPQ (**F**) intensity related measurements in *vcpALS* versus *comALS* classifiers. Linear mixed effects analysis of the relationship between the type of classifiers (*comALS* versus *vcpALS*) and the relative contribution of the measurement categories to account for idiosyncratic variation due to classifiers. Data shown as box plots in which the centre line is the median, limits are the interquartile range and whiskers are the minimum and maximum. Green = *vcpALS* classifiers; grey = *comALS* classifiers.

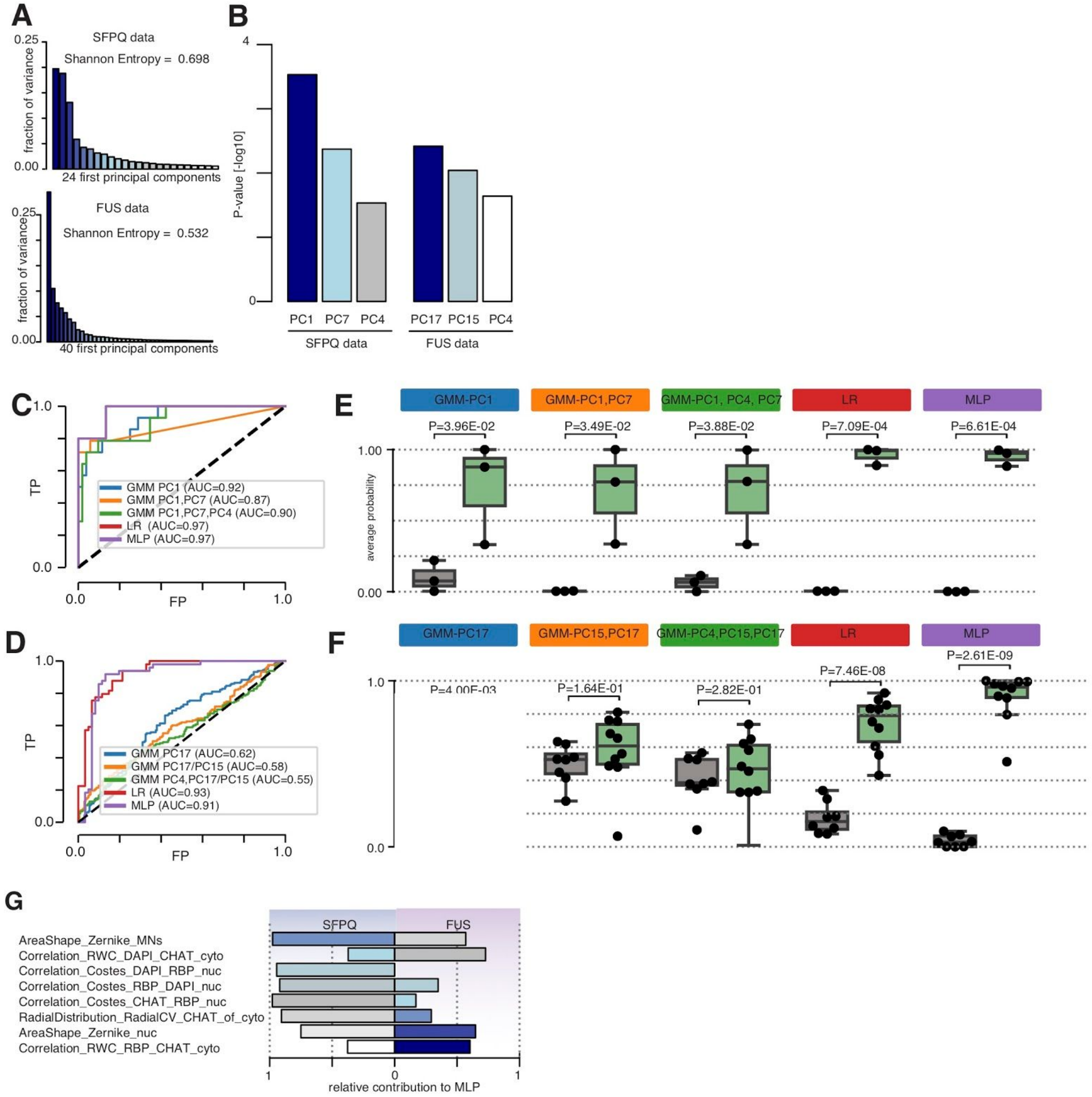

Supplementary Figure 5 | (**A**) Fraction of explained variance captured by the first 24 and 40 principal components that captures 90% of the signal in SFPQ and FUS data respectively. (**B**) Barplots showing the association between principal components and ALS in SFPQ (*left)* and FUS (*right*) data. Linear mixed effects analysis of the relationship between ALS phenotype and each of the 24 and 40 first principal components to account for idiosyncratic variation due to individuals shows significant association of PC1, PC4 and PC7 and ALS in SFPQ data, and association between

PC4, PC15 and PC17 with ALS in FUS data. (**C**,**D**) Performance analysis of each classifier in SFPQ data (**C**) or FUS data (**D**) in their ability to discriminate sALS MNs from healthy MNs using receiver operating characteristic (ROC) curves and aurea under the curves (AUC). (**E**, **F**) The ability for each clustering algorithm to detect sALS effect is assessed by comparing the disease probabilities of sALS group with those of the control group. The disease probability of each individual is obtained by using the mean probabilities of its cells to be sick according to individual classifiers in SFPQ data (**B**) or FUS data (**F**). Data shown as box plots in which the centre line is the median, limits are the interquartile range and whiskers are the minimum and maximum. Dots are the individual disease profile. *P*-values obtained from Welch's t test. (**G**) Barplots showing the relative contribution of the top five measurements in MLP in either SFPQ or FUS post-mortem tissue data. Zernike moments either in the nucleus or the whole MNs contribute largely to both MLP classifiers. Bars are color-coded according to the ranking in contribution for the given classifier, from dark blue to white for high to low ranking.

SUPPEMENTARY TABLES

imageID	mutation	RBP1	RBP2	animal	origin	Lab	pairs
**4G10D1**	SOD1	SFPQ	FUS	4G10	Lab_2	Lab_2	SFPQ_FUS
**4G10D2**	SOD1	SFPQ	FUS	4G10	Lab_2	Lab_2	SFPQ_FUS
**4G12D1**	SOD1	SFPQ	FUS	4G12	Lab_2	Lab_2	SFPQ_FUS
**4G12D2**	SOD1	SFPQ	FUS	4G12	Lab_2	Lab_2	SFPQ_FUS
**4G13D1**	CTRL	SFPQ	FUS	4G13	Lab_2	Lab_2	SFPQ_FUS
**4G13D2**	CTRL	SFPQ	FUS	4G13	Lab_2	Lab_2	SFPQ_FUS
**4G13D3**	CTRL	SFPQ	FUS	4G13	Lab_2	Lab_2	SFPQ_FUS
**4G14D1**	SOD1	SFPQ	FUS	4G14	Lab_2	Lab_2	SFPQ_FUS
**4G14D2**	SOD1	SFPQ	FUS	4G14	Lab_2	Lab_2	SFPQ_FUS
**4G14D3**	SOD1	SFPQ	FUS	4G14	Lab_2	Lab_2	SFPQ_FUS
**4G15D1**	CTRL	SFPQ	FUS	4G15	Lab_2	Lab_2	SFPQ_FUS
**4G15D2**	CTRL	SFPQ	FUS	4G15	Lab_2	Lab_2	SFPQ_FUS

**4G16D1**	SOD1	SFPQ	FUS	4G16	Lab_2	Lab_2	SFPQ_FUS
**4G16D2**	SOD1	SFPQ	FUS	4G16	Lab_2	Lab_2	SFPQ_FUS
**4G16D3**	SOD1	SFPQ	FUS	4G16	Lab_2	Lab_2	SFPQ_FUS
**4G9D1**	CTRL	SFPQ	FUS	4G9	Lab_2	Lab_2	SFPQ_FUS
**4G9D2**	CTRL	SFPQ	FUS	4G9	Lab_2	Lab_2	SFPQ_FUS
**4G9D3**	CTRL	SFPQ	FUS	4G9	Lab_2	Lab_2	SFPQ_FUS
**VCP1AFITC**	VCP	SFPQ	FUS	VCP1	Lab_1	Lab_1	SFPQ_FUS
**VCP1BCY5**	VCP	SFPQ	FUS	VCP1	Lab_1	Lab_1	SFPQ_FUS
**VCP1BFITC**	VCP	SFPQ	FUS	VCP1	Lab_1	Lab_1	SFPQ_FUS
**VCP1CFITC**	VCP	SFPQ	FUS	VCP1	Lab_1	Lab_1	SFPQ_FUS
**VCP1DFITC**	VCP	SFPQ	FUS	VCP1	Lab_1	Lab_1	SFPQ_FUS
**VCP1EFITC**	VCP	SFPQ	FUS	VCP1	Lab_1	Lab_1	SFPQ_FUS
**VCP2A**	VCP	SFPQ	FUS	VCP2	Lab_1	Lab_1	SFPQ_FUS
**VCP2AMODIFIED**	VCP	SFPQ	FUS	VCP2	Lab_1	Lab_1	SFPQ_FUS
**VCP2BFITIC**	VCP	SFPQ	FUS	VCP2	Lab_1	Lab_1	SFPQ_FUS
**VCP2BMODIF**	VCP	SFPQ	FUS	VCP2	Lab_1	Lab_1	SFPQ_FUS
**VCP2CFITIC**	VCP	SFPQ	FUS	VCP2	Lab_1	Lab_1	SFPQ_FUS
**VCP2DFITC**	VCP	SFPQ	FUS	VCP2	Lab_1	Lab_1	SFPQ_FUS
**VCP2EFITC**	VCP	SFPQ	FUS	VCP2	Lab_1	Lab_1	SFPQ_FUS
**VCP2FFITC**	VCP	SFPQ	FUS	VCP2	Lab_1	Lab_1	SFPQ_FUS
**VCP3A**	VCP	SFPQ	FUS	VCP3	Lab_1	Lab_1	SFPQ_FUS
**VCP3B**	VCP	SFPQ	FUS	VCP3	Lab_1	Lab_1	SFPQ_FUS
**VCP3C**	VCP	SFPQ	FUS	VCP3	Lab_1	Lab_1	SFPQ_FUS
**VCP3D**	VCP	SFPQ	FUS	VCP3	Lab_1	Lab_1	SFPQ_FUS
**VCP3E**	VCP	SFPQ	FUS	VCP3	Lab_1	Lab_1	SFPQ_FUS
**VCP3F**	VCP	SFPQ	FUS	VCP3	Lab_1	Lab_1	SFPQ_FUS

Table S1 | List of images used for FUS and SFPQ cellular localisation in (Tyzack *et al*, 2019; Luisier *et al*, 2018); mouse data.

Batch	ID	ImageID	RBP	Group	imageID	frame	round	patient	rbp1
**1TG**	1TG1	TG1AL1-ChAT.tif	FUS	CTRL	1TG1AL1	AL1	AL	1TG1	FUS
**1TG**	1TG1	TG1AL2-ChAT.tif	FUS	CTRL	1TG1AL2	AL2	AL	1TG1	FUS
**1TG**	1TG1	TG1AL3-ChAT.tif	FUS	CTRL	1TG1AL3	AL3	AL	1TG1	FUS
**1TG**	1TG1	TG1AL4-ChAT.tif	FUS	CTRL	1TG1AL4	AL4	AL	1TG1	FUS
**1TG**	1TG1	TG1AL5-ChAT.tif	FUS	CTRL	1TG1AL5	AL5	AL	1TG1	FUS
**1TG**	1TG1	TG1AM1-ChAT.tif	FUS	CTRL	1TG1AM1	AM1	AM	1TG1	FUS
**1TG**	1TG1	TG1AM2-ChAT.tif	FUS	CTRL	1TG1AM2	AM2	AM	1TG1	FUS
**1TG**	1TG1	TG1AM3-ChAT.tif	FUS	CTRL	1TG1AM3	AM3	AM	1TG1	FUS
**1TG**	1TG1	TG1AM4-ChAT.tif	FUS	CTRL	1TG1AM4	AM4	AM	1TG1	FUS
**1TG**	1TG1	TG1AR1-ChAT.tif	FUS	CTRL	1TG1AR1	AR1	AR	1TG1	FUS
**1TG**	1TG1	TG1AR2-ChAT.tif	FUS	CTRL	1TG1AR2	AR2	AR	1TG1	FUS
**1TG**	1TG1	TG1AR3-ChAT.tif	FUS	CTRL	1TG1AR3	AR3	AR	1TG1	FUS
**1TG**	1TG1	TG1AR4-ChAT.tif	FUS	CTRL	1TG1AR4	AR4	AR	1TG1	FUS

**1TG**	1TG2	TG2A-ChAT.tif	FUS	CTRL	1TG2A	A	A	1TG2	FUS
**1TG**	1TG2	TG2AI1-ChAT.tif	FUS	CTRL	1TG2AI1	AI1	AI	1TG2	FUS
**1TG**	1TG2	TG2AL3-ChAT.tif	FUS	CTRL	1TG2AL3	AL3	AL	1TG2	FUS
**1TG**	1TG2	TG2AL4-ChAT.tif	FUS	CTRL	1TG2AL4	AL4	AL	1TG2	FUS
**1TG**	1TG2	TG2AM1-ChAT.tif	FUS	CTRL	1TG2AM1	AM1	AM	1TG2	FUS
**1TG**	1TG2	TG2AM2-ChAT.tif	FUS	CTRL	1TG2AM2	AM2	AM	1TG2	FUS
**1TG**	1TG2	TG2AM23-ChAT.tif	FUS	CTRL	1TG2AM23	AM23	AM	1TG2	FUS
**1TG**	1TG2	TG2AM4-ChAT.tif	FUS	CTRL	1TG2AM4	AM4	AM	1TG2	FUS
**1TG**	1TG2	TG2AM5-ChAT.tif	FUS	CTRL	1TG2AM5	AM5	AM	1TG2	FUS
**1TG**	1TG2	TG2AR1-ChAT.tif	FUS	CTRL	1TG2AR1	AR1	AR	1TG2	FUS
**1TG**	1TG2	TG2AR2-ChAT.tif	FUS	CTRL	1TG2AR2	AR2	AR	1TG2	FUS
**1TG**	1TG2	TG2AR3-ChAT.tif	FUS	CTRL	1TG2AR3	AR3	AR	1TG2	FUS
**1TG**	1TG2	TG2AR4-ChAT.tif	FUS	CTRL	1TG2AR4	AR4	AR	1TG2	FUS
**1TG**	1TG2	TG2AR5-ChAT.tif	FUS	CTRL	1TG2AR5	AR5	AR	1TG2	FUS
**1TG**	1TG2	TG2AR6-ChAT.tif	FUS	CTRL	1TG2AR6	AR6	AR	1TG2	FUS
**1TG**	1TG2	TG2AR7-ChAT.tif	FUS	CTRL	1TG2AR7	AR7	AR	1TG2	FUS
**1TG**	1TG2	TG2AR8-ChAT.tif	FUS	CTRL	1TG2AR8	AR8	AR	1TG2	FUS
**1TG**	1TG3	TG3AL1-ChAT.tif	FUS	sALS	1TG3AL1	AL1	AL	1TG3	FUS
**1TG**	1TG3	TG3AL2-ChAT.tif	FUS	sALS	1TG3AL2	AL2	AL	1TG3	FUS
**1TG**	1TG3	TG3AL3-ChAT.tif	FUS	sALS	1TG3AL3	AL3	AL	1TG3	FUS
**1TG**	1TG3	TG3AL4-ChAT.tif	FUS	sALS	1TG3AL4	AL4	AL	1TG3	FUS
**1TG**	1TG3	TG3Am1-ChAT.tif	FUS	sALS	1TG3Am1	Am1	Am	1TG3	FUS
**1TG**	1TG3	TG3AM2-ChAT.tif	FUS	sALS	1TG3AM2	AM2	AM	1TG3	FUS
**1TG**	1TG3	TG3AM3-ChAT.tif	FUS	sALS	1TG3AM3	AM3	AM	1TG3	FUS
**1TG**	1TG3	TG3AM4-ChAT.tif	FUS	sALS	1TG3AM4	AM4	AM	1TG3	FUS
**1TG**	1TG3	TG3AM5-ChAT.tif	FUS	sALS	1TG3AM5	AM5	AM	1TG3	FUS
**1TG**	1TG3	TG3AM6-ChAT.tif	FUS	sALS	1TG3AM6	AM6	AM	1TG3	FUS
**1TG**	1TG3	TG3AM7-ChAT.tif	FUS	sALS	1TG3AM7	AM7	AM	1TG3	FUS
**1TG**	1TG3	TG3AR1-ChAT.tif	FUS	sALS	1TG3AR1	AR1	AR	1TG3	FUS
**1TG**	1TG3	TG3AR2-ChAT.tif	FUS	sALS	1TG3AR2	AR2	AR	1TG3	FUS

**1TG**	1TG3	TG3AR3-ChAT.tif	FUS	sALS	1TG3AR3	AR3	AR	1TG3	FUS
**1TG**	1TG3	TG3AR4-ChAT.tif	FUS	sALS	1TG3AR4	AR4	AR	1TG3	FUS
**1TG**	1TG4	TG4L1-ChAT.tif	FUS	sALS	1TG4L1	L1	L	1TG4	FUS
**1TG**	1TG4	TG4L2-ChAT.tif	FUS	sALS	1TG4L2	L2	L	1TG4	FUS
**1TG**	1TG4	TG4L3-ChAT.tif	FUS	sALS	1TG4L3	L3	L	1TG4	FUS
**1TG**	1TG4	TG4L4-ChAT.tif	FUS	sALS	1TG4L4	L4	L	1TG4	FUS
**1TG**	1TG4	TG4M1-ChAT.tif	FUS	sALS	1TG4M1	M1	M	1TG4	FUS
**1TG**	1TG4	TG4M2-ChAT.tif	FUS	sALS	1TG4M2	M2	M	1TG4	FUS
**1TG**	1TG4	TG4M3-ChAT.tif	FUS	sALS	1TG4M3	M3	M	1TG4	FUS
**1TG**	1TG4	TG4M4-ChAT.tif	FUS	sALS	1TG4M4	M4	M	1TG4	FUS
**1TG**	1TG4	TG4M5-ChAT.tif	FUS	sALS	1TG4M5	M5	M	1TG4	FUS
**1TG**	1TG4	TG4R1-ChAT.tif	FUS	sALS	1TG4R1	R1	R	1TG4	FUS
**1TG**	1TG4	TG4R2-ChAT.tif	FUS	sALS	1TG4R2	R2	R	1TG4	FUS
**1TG**	1TG4	TG4R3-ChAT.tif	FUS	sALS	1TG4R3	R3	R	1TG4	FUS
**1TG**	1TG4	TG4R4-ChAT.tif	FUS	sALS	1TG4R4	R4	R	1TG4	FUS
**1TG**	1TG4	TG4R5-ChAT.tif	FUS	sALS	1TG4R5	R5	R	1TG4	FUS
**1TG**	1TG5	TG5Al1-ChAT.tif	FUS	sALS	1TG5Al1	Al1	Al	1TG5	FUS
**1TG**	1TG5	TG5AL2-ChAT.tif	FUS	sALS	1TG5AL2	AL2	AL	1TG5	FUS
**1TG**	1TG5	TG5AM1-ChAT.tif	FUS	sALS	1TG5AM1	AM1	AM	1TG5	FUS
**1TG**	1TG5	TG5AM2-ChAT.tif	FUS	sALS	1TG5AM2	AM2	AM	1TG5	FUS
**1TG**	1TG5	TG5AM3-ChAT.tif	FUS	sALS	1TG5AM3	AM3	AM	1TG5	FUS
**1TG**	1TG5	TG5AM4-ChAT.tif	FUS	sALS	1TG5AM4	AM4	AM	1TG5	FUS
**1TG**	1TG5	TG5AR1-ChAT.tif	FUS	sALS	1TG5AR1	AR1	AR	1TG5	FUS
**1TG**	1TG5	TG5AR2-ChAT.tif	FUS	sALS	1TG5AR2	AR2	AR	1TG5	FUS
**1TG**	1TG5	TG5AR3-ChAT.tif	FUS	sALS	1TG5AR3	AR3	AR	1TG5	FUS
**1TG**	1TG5	TG5AR4-ChAT.tif	FUS	sALS	1TG5AR4	AR4	AR	1TG5	FUS
**2TG**	2TG4	2TG4A1-ChAT.tif	FUS	sALS	2TG4A1	A1	A	2TG4	FUS
**2TG**	2TG4	2TG4A10-ChAT.tif	FUS	sALS	2TG4A10	A10	A	2TG4	FUS
**2TG**	2TG4	2TG4A2-ChAT.tif	FUS	sALS	2TG4A2	A2	A	2TG4	FUS
**2TG**	2TG4	2TG4A3-ChAT.tif	FUS	sALS	2TG4A3	A3	A	2TG4	FUS
**2TG**	2TG4	2TG4A4-ChAT.tif	FUS	sALS	2TG4A4	A4	A	2TG4	FUS

**2TG**	2TG4	2TG4A5-ChAT.tif	FUS	sALS	2TG4A5	A5	A	2TG4	FUS
**2TG**	2TG4	2TG4A6-ChAT.tif	FUS	sALS	2TG4A6	A6	A	2TG4	FUS
**2TG**	2TG4	2TG4A7-ChAT.tif	FUS	sALS	2TG4A7	A7	A	2TG4	FUS
**2TG**	2TG4	2TG4A8-ChAT.tif	FUS	sALS	2TG4A8	A8	A	2TG4	FUS
**2TG**	2TG4	2TG4A9-ChAT.tif	FUS	sALS	2TG4A9	A9	A	2TG4	FUS
**2TG**	2TG5	2TG5A1-ChAT.tif	FUS	sALS	2TG5A1	A1	A	2TG5	FUS
**2TG**	2TG5	2TG5A2-ChAT.tif	FUS	sALS	2TG5A2	A2	A	2TG5	FUS
**2TG**	2TG5	2TG5A3-ChAT.tif	FUS	sALS	2TG5A3	A3	A	2TG5	FUS
**2TG**	2TG5	2TG5A4-ChAT.tif	FUS	sALS	2TG5A4	A4	A	2TG5	FUS
**2TG**	2TG5	2TG5A5-ChAT.tif	FUS	sALS	2TG5A5	A5	A	2TG5	FUS
**2TG**	2TG5	2TG5A6-ChAT.tif	FUS	sALS	2TG5A6	A6	A	2TG5	FUS
**2TG**	2TG5	2TG5A7-ChAT.tif	FUS	sALS	2TG5A7	A7	A	2TG5	FUS
**2TG**	2TG6	2TG6A1-ChAT.tif	FUS	sALS	2TG6A1	A1	A	2TG6	FUS
**2TG**	2TG6	2TG6A2-ChAT.tif	FUS	sALS	2TG6A2	A2	A	2TG6	FUS
**2TG**	2TG6	2TG6A3-ChAT.tif	FUS	sALS	2TG6A3	A3	A	2TG6	FUS
**2TG**	2TG6	2TG6A4-ChAT.tif	FUS	sALS	2TG6A4	A4	A	2TG6	FUS
**2TG**	2TG6	2TG6A5-ChAT.tif	FUS	sALS	2TG6A5	A5	A	2TG6	FUS
**2TG**	2TG6	2TG6A6-ChAT.tif	FUS	sALS	2TG6A6	A6	A	2TG6	FUS
**2TG**	2TG6	2TG6A7-ChAT.tif	FUS	sALS	2TG6A7	A7	A	2TG6	FUS
**2TG**	2TG6	2TG6A8-ChAT.tif	FUS	sALS	2TG6A8	A8	A	2TG6	FUS
**3TG**	3TG1	3TG1A1-ChAT.tif	FUS	CTRL	3TG1A1	A1	A	3TG1	FUS
**3TG**	3TG1	3TG1A2-ChAT.tif	FUS	CTRL	3TG1A2	A2	A	3TG1	FUS
**3TG**	3TG1	3TG1A3-ChAT.tif	FUS	CTRL	3TG1A3	A3	A	3TG1	FUS
**3TG**	3TG1	3TG1A4-ChAT.tif	FUS	CTRL	3TG1A4	A4	A	3TG1	FUS
**3TG**	3TG1	3TG1A5-ChAT.tif	FUS	CTRL	3TG1A5	A5	A	3TG1	FUS
**3TG**	3TG1	3TG1A6-ChAT.tif	FUS	CTRL	3TG1A6	A6	A	3TG1	FUS
**3TG**	3TG2	3TG2A1-ChAT.tif	FUS	CTRL	3TG2A1	A1	A	3TG2	FUS
**3TG**	3TG2	3TG2A2-ChAT.tif	FUS	CTRL	3TG2A2	A2	A	3TG2	FUS
**3TG**	3TG2	3TG2A3-ChAT.tif	FUS	CTRL	3TG2A3	A3	A	3TG2	FUS
**3TG**	3TG2	3TG2A4-ChAT.tif	FUS	CTRL	3TG2A4	A4	A	3TG2	FUS
**3TG**	3TG2	3TG2A5-ChAT.tif	FUS	CTRL	3TG2A5	A5	A	3TG2	FUS

**3TG**	3TG2	3TG2A6-ChAT.tif	FUS	CTRL	3TG2A6	A6	A	3TG2	FUS
**3TG**	3TG2	3TG2A7-ChAT.tif	FUS	CTRL	3TG2A7	A7	A	3TG2	FUS
**3TG**	3TG3	3TG3A1-ChAT.tif	FUS	CTRL	3TG3A1	A1	A	3TG3	FUS
**3TG**	3TG3	3TG3A2-ChAT.tif	FUS	CTRL	3TG3A2	A2	A	3TG3	FUS
**3TG**	3TG3	3TG3A3-ChAT.tif	FUS	CTRL	3TG3A3	A3	A	3TG3	FUS
**3TG**	3TG3	3TG3A4-ChAT.tif	FUS	CTRL	3TG3A4	A4	A	3TG3	FUS
**3TG**	3TG3	3TG3A5-ChAT.tif	FUS	CTRL	3TG3A5	A5	A	3TG3	FUS
**3TG**	3TG3	3TG3A6-ChAT.tif	FUS	CTRL	3TG3A6	A6	A	3TG3	FUS
**3TG**	3TG3	3TG3A7-ChAT.tif	FUS	CTRL	3TG3A7	A7	A	3TG3	FUS
**3TG**	3TG3	3TG3A8	FUS	CTRL	3TG3A8	A8	A	3TG3	FUS
**3TG**	3TG4	3TG4A1-ChAT.tif	FUS	sALS	3TG4A1	A1	A	3TG4	FUS
**3TG**	3TG4	3TG4A2-ChAT.tif	FUS	sALS	3TG4A2	A2	A	3TG4	FUS
**3TG**	3TG4	3TG4A3-ChAT.tif	FUS	sALS	3TG4A3	A3	A	3TG4	FUS
**3TG**	3TG4	3TG4A4-ChAT.tif	FUS	sALS	3TG4A4	A4	A	3TG4	FUS
**3TG**	3TG4	3TG4A5-ChAT.tif	FUS	sALS	3TG4A5	A5	A	3TG4	FUS
**3TG**	3TG4	3TG4A6-ChAT.tif	FUS	sALS	3TG4A6	A6	A	3TG4	FUS
**3TG**	3TG4	3TG4A7-ChAT.tif	FUS	sALS	3TG4A7	A7	A	3TG4	FUS
**3TG**	3TG4	3TG4A8-ChAT.tif	FUS	sALS	3TG4A8	A8	A	3TG4	FUS
**3TG**	3TG5	3TG5A1	FUS	sALS	3TG5A1	A1	A	3TG5	FUS
**3TG**	3TG5	3TG5A2	FUS	sALS	3TG5A2	A2	A	3TG5	FUS
**3TG**	3TG5	3TG5A3	FUS	sALS	3TG5A3	A3	A	3TG5	FUS
**3TG**	3TG5	3TG5A4	FUS	sALS	3TG5A4	A4	A	3TG5	FUS
**3TG**	3TG5	3TG5A5	FUS	sALS	3TG5A5	A5	A	3TG5	FUS
**3TG**	3TG5	3TG5A6	FUS	sALS	3TG5A6	A6	A	3TG5	FUS
**3TG**	3TG6	3TG6A1	FUS	sALS	3TG6A1	A1	A	3TG6	FUS
**3TG**	3TG6	3TG6A2	FUS	sALS	3TG6A2	A2	A	3TG6	FUS
**3TG**	3TG6	3TG6A3	FUS	sALS	3TG6A3	A3	A	3TG6	FUS
**3TG**	3TG6	3TG6A4	FUS	sALS	3TG6A4	A4	A	3TG6	FUS
**3TG**	3TG6	3TG6A5	FUS	sALS	3TG6A5	A5	A	3TG6	FUS
**3TG**	3TG6	3TG6A6	FUS	sALS	3TG6A6	A6	A	3TG6	FUS
**3TG**	3TG6	3TG6A7	FUS	sALS	3TG6A7	A7	A	3TG6	FUS

**3TG**	3TG6	3TG6A8	FUS	sALS	3TG6A8	A8	A	3TG6	FUS
**4KI**	4Ki1	4KI1B1L-ChAT.tif	FUS	CTRL	4Ki1B1L	B1L	BL	4Ki1	FUS
**4KI**	4Ki1	4KI1B1M-ChAT.tif	FUS	CTRL	4Ki1B1M	B1M	BM	4Ki1	FUS
**4KI**	4Ki1	4KI1B1R-ChAT.tif	FUS	CTRL	4Ki1B1R	B1R	BR	4Ki1	FUS
**4KI**	4Ki1	4KI1B2L-ChAT.tif	FUS	CTRL	4Ki1B2L	B2L	BL	4Ki1	FUS
**4KI**	4Ki1	4KI1B2M-ChAT.tif	FUS	CTRL	4Ki1B2M	B2M	BM	4Ki1	FUS
**4KI**	4Ki1	4KI1B2R-ChAT.tif	FUS	CTRL	4Ki1B2R	B2R	BR	4Ki1	FUS
**4KI**	4Ki1	4KI1B3L-ChAT.tif	FUS	CTRL	4Ki1B3L	B3L	BL	4Ki1	FUS
**4KI**	4Ki1	4KI1B3M-ChAT.tif	FUS	CTRL	4Ki1B3M	B3M	BM	4Ki1	FUS
**4KI**	4Ki1	4KI1B3R-ChAT.tif	FUS	CTRL	4Ki1B3R	B3R	BR	4Ki1	FUS
**4KI**	4Ki1	4KI1B4L-ChAT.tif	FUS	CTRL	4Ki1B4L	B4L	BL	4Ki1	FUS
**4KI**	4Ki1	4KI1B4R-ChAT.tif	FUS	CTRL	4Ki1B4R	B4R	BR	4Ki1	FUS
**4KI**	4Ki1	4KI1B5R-ChAT.tif	FUS	CTRL	4Ki1B5R	B5R	BR	4Ki1	FUS
**4KI**	4Ki1	4KI1B6R	FUS	CTRL	4Ki1B6R	B6R	BR	4Ki1	FUS
**4KI**	4Ki2	4KI2B1R-ChAT.tif	FUS	CTRL	4Ki2B1R	B1R	BR	4Ki2	FUS
**4KI**	4Ki2	4KI2B2M-ChAT.tif	FUS	CTRL	4Ki2B2M	B2M	BM	4Ki2	FUS
**4KI**	4Ki2	4KI2B2R-ChAT.tif	FUS	CTRL	4Ki2B2R	B2R	BR	4Ki2	FUS
**4KI**	4Ki2	4KI2B3M-ChAT.tif	FUS	CTRL	4Ki2B3M	B3M	BM	4Ki2	FUS
**4KI**	4Ki2	4KI2B4R-ChAT.tif	FUS	CTRL	4Ki2B4R	B4R	BR	4Ki2	FUS
**4KI**	4Ki2	4KI2B1m	FUS	CTRL	4Ki2B1m	B1m	Bm	4Ki2	FUS
**4KI**	4Ki2	4KI2B3R	FUS	CTRL	4Ki2B3R	B3R	BR	4Ki2	FUS
**4KI**	4Ki3	4KI3B1L-ChAT.tif	FUS	CTRL	4Ki3B1L	B1L	BL	4Ki3	FUS
**4KI**	4Ki3	4KI3B1M-ChAT.tif	FUS	CTRL	4Ki3B1M	B1M	BM	4Ki3	FUS
**4KI**	4Ki3	4KI3B1R-ChAT.tif	FUS	CTRL	4Ki3B1R	B1R	BR	4Ki3	FUS
**4KI**	4Ki3	4KI3B2L-ChAT.tif	FUS	CTRL	4Ki3B2L	B2L	BL	4Ki3	FUS
**4KI**	4Ki3	4KI3B2M-ChAT.tif	FUS	CTRL	4Ki3B2M	B2M	BM	4Ki3	FUS
**4KI**	4Ki3	4KI3B2R-ChAT.tif	FUS	CTRL	4Ki3B2R	B2R	BR	4Ki3	FUS
**4KI**	4Ki3	4KI3B3L-ChAT.tif	FUS	CTRL	4Ki3B3L	B3L	BL	4Ki3	FUS
**4KI**	4Ki3	4KI3B3M-ChAT.tif	FUS	CTRL	4Ki3B3M	B3M	BM	4Ki3	FUS
**4KI**	4Ki3	4KI3B3R-ChAT.tif	FUS	CTRL	4Ki3B3R	B3R	BR	4Ki3	FUS
**4KI**	4Ki3	4KI3B4M-ChAT.tif	FUS	CTRL	4Ki3B4M	B4M	BM	4Ki3	FUS

**4KI**	4Ki3	4KI3B4R	FUS	CTRL	4Ki3B4R	B4R	BR	4Ki3	FUS
**4KI**	4Ki3	4KI3B5R	FUS	CTRL	4Ki3B5R	B5R	BR	4Ki3	FUS
**4KI**	4Ki4	4KI4B1M-ChAT.tif	FUS	sALS	4Ki4B1M	B1M	BM	4Ki4	FUS
**4KI**	4Ki4	4KI4B1R-ChAT.tif	FUS	sALS	4Ki4B1R	B1R	BR	4Ki4	FUS
**4KI**	4Ki4	4KI4B2R-ChAT.tif	FUS	sALS	4Ki4B2R	B2R	BR	4Ki4	FUS
**4KI**	4Ki4	4KI4B3R-ChAT.tif	FUS	sALS	4Ki4B3R	B3R	BR	4Ki4	FUS
**4KI**	4Ki4	4KI4B4R-ChAT.tif	FUS	sALS	4Ki4B4R	B4R	BR	4Ki4	FUS
**4KI**	4Ki4	4KI4BM1	FUS	sALS	4Ki4BM1	BM1	BM	4Ki4	FUS
**4KI**	4Ki4	4KI4BM2	FUS	sALS	4Ki4BM2	BM2	BM	4Ki4	FUS
**4KI**	4Ki5	4KI5B1L-ChAT.tif	FUS	sALS	4Ki5B1L	B1L	BL	4Ki5	FUS
**4KI**	4Ki5	4KI5B1M-ChAT.tif	FUS	sALS	4Ki5B1M	B1M	BM	4Ki5	FUS
**4KI**	4Ki5	4KI5B1R-ChAT.tif	FUS	sALS	4Ki5B1R	B1R	BR	4Ki5	FUS
**4KI**	4Ki5	4KI5B2M-ChAT.tif	FUS	sALS	4Ki5B2M	B2M	BM	4Ki5	FUS
**4KI**	4Ki5	4KI5B2R-ChAT.tif	FUS	sALS	4Ki5B2R	B2R	BR	4Ki5	FUS
**4KI**	4Ki6	4KI6B2R-ChAT.tif	FUS	sALS	4Ki6B2R	B2R	BR	4Ki6	FUS
**4KI**	4Ki6	4KI6B3R-ChAT.tif	FUS	sALS	4Ki6B3R	B3R	BR	4Ki6	FUS
**4KI**	4Ki6	4KI6B4R-ChAT.tif	FUS	sALS	4Ki6B4R	B4R	BR	4Ki6	FUS
**4KI**	4Ki6	4KI6BL1lsm-ChAT.tif	FUS	sALS	4Ki6BL1lsm	BL1lsm	BLlsm	4Ki6	FUS
**4KI**	4Ki6	4KI6BL2-ChAT.tif	FUS	sALS	4Ki6BL2	BL2	BL	4Ki6	FUS
**4KI**	4Ki6	4KI6BL3-ChAT.tif	FUS	sALS	4Ki6BL3	BL3	BL	4Ki6	FUS
**4KI**	4Ki6	4KI6BL4-ChAT.tif	FUS	sALS	4Ki6BL4	BL4	BL	4Ki6	FUS
**4KI**	4Ki6	4KI6BM2-ChAT.tif	FUS	sALS	4Ki6BM2	BM2	BM	4Ki6	FUS
**4KI**	4Ki6	4KI6BM3-ChAT.tif	FUS	sALS	4Ki6BM3	BM3	BM	4Ki6	FUS
**4KI**	4Ki6	4KI6BM4-ChAT.tif	FUS	sALS	4Ki6BM4	BM4	BM	4Ki6	FUS
**4KI**	4Ki6	4KI6BM5-ChAT.tif	FUS	sALS	4Ki6BM5	BM5	BM	4Ki6	FUS
**4KI**	4Ki6	4KI6B1R	FUS	sALS	4Ki6B1R	B1R	BR	4Ki6	FUS
**4KI**	4Ki6	4KI6BM1	FUS	sALS	4Ki6BM1	BM1	BM	4Ki6	FUS
**4KI**	4Ki1	2ki1Dc1-ChAT.tif	SFPQ	CTRL	4Ki1Dc1	Dc1	Dc	4Ki1	SFPQ
**4KI**	4Ki1	2ki1Dc3-ChAT.tif	SFPQ	CTRL	4Ki1Dc3	Dc3	Dc	4Ki1	SFPQ
**4KI**	4Ki1	2ki1Dl1-ChAT.tif	SFPQ	CTRL	4Ki1Dl1	Dl1	Dl	4Ki1	SFPQ

**4KI**	4Ki1	2ki1Dr1-ChAT.tif	SFPQ	CTRL	4Ki1Dr1	Dr1	Dr	4Ki1	SFPQ
**4KI**	4Ki1	2ki1Dr2-ChAT.tif	SFPQ	CTRL	4Ki1Dr2	Dr2	Dr	4Ki1	SFPQ
**4KI**	4Ki1	2ki1Dc2	SFPQ	CTRL	4Ki1Dc2	Dc2	Dc	4Ki1	SFPQ
**4KI**	4Ki2	2ki2Dc1-ChAT.tif	SFPQ	CTRL	4Ki2Dc1	Dc1	Dc	4Ki2	SFPQ
**4KI**	4Ki2	2ki2Dc3-ChAT.tif	SFPQ	CTRL	4Ki2Dc3	Dc3	Dc	4Ki2	SFPQ
**4KI**	4Ki2	2ki2Dr2-ChAT.tif	SFPQ	CTRL	4Ki2Dr2	Dr2	Dr	4Ki2	SFPQ
**4KI**	4Ki2	2ki2Dr3-ChAT.tif	SFPQ	CTRL	4Ki2Dr3	Dr3	Dr	4Ki2	SFPQ
**4KI**	4Ki2	2ki2Dc2	SFPQ	CTRL	4Ki2Dc2	Dc2	Dc	4Ki2	SFPQ
**4KI**	4Ki3	2ki3DC1-ChAT.tif	SFPQ	CTRL	4Ki3DC1	DC1	DC	4Ki3	SFPQ
**4KI**	4Ki3	2ki3Dc3-ChAT.tif	SFPQ	CTRL	4Ki3Dc3	Dc3	Dc	4Ki3	SFPQ
**4KI**	4Ki3	2ki3Dc4-ChAT.tif	SFPQ	CTRL	4Ki3Dc4	Dc4	Dc	4Ki3	SFPQ
**4KI**	4Ki3	2ki3Dc5-ChAT.tif	SFPQ	CTRL	4Ki3Dc5	Dc5	Dc	4Ki3	SFPQ
**4KI**	4Ki3	2ki3Dl1-ChAT.tif	SFPQ	CTRL	4Ki3Dl1	Dl1	Dl	4Ki3	SFPQ
**4KI**	4Ki3	2ki3Dl2-ChAT.tif	SFPQ	CTRL	4Ki3Dl2	Dl2	Dl	4Ki3	SFPQ
**4KI**	4Ki3	2ki3Dl3-ChAT.tif	SFPQ	CTRL	4Ki3Dl3	Dl3	Dl	4Ki3	SFPQ
**4KI**	4Ki3	2ki3Dr1-ChAT.tif	SFPQ	CTRL	4Ki3Dr1	Dr1	Dr	4Ki3	SFPQ
**4KI**	4Ki3	2ki3Dr2-ChAT.tif	SFPQ	CTRL	4Ki3Dr2	Dr2	Dr	4Ki3	SFPQ
**4KI**	4Ki3	2ki3Dc2	SFPQ	CTRL	4Ki3Dc2	Dc2	Dc	4Ki3	SFPQ
**4KI**	4Ki4	2ki4Dc1-ChAT.tif	SFPQ	sALS	4Ki4Dc1	Dc1	Dc	4Ki4	SFPQ
**4KI**	4Ki4	2ki4Dc2-ChAT.tif	SFPQ	sALS	4Ki4Dc2	Dc2	Dc	4Ki4	SFPQ
**4KI**	4Ki4	2ki4Dc3-ChAT.tif	SFPQ	sALS	4Ki4Dc3	Dc3	Dc	4Ki4	SFPQ
**4KI**	4Ki4	2ki4Dl1-ChAT.tif	SFPQ	sALS	4Ki4Dl1	Dl1	Dl	4Ki4	SFPQ
**4KI**	4Ki4	2ki4Dl2-ChAT.tif	SFPQ	sALS	4Ki4Dl2	Dl2	Dl	4Ki4	SFPQ
**4KI**	4Ki4	2ki4Dr1-ChAT.tif	SFPQ	sALS	4Ki4Dr1	Dr1	Dr	4Ki4	SFPQ
**4KI**	4Ki4	2ki4Dr3-ChAT.tif	SFPQ	sALS	4Ki4Dr3	Dr3	Dr	4Ki4	SFPQ
**4KI**	4Ki4	2ki4Dr4-ChAT.tif	SFPQ	sALS	4Ki4Dr4	Dr4	Dr	4Ki4	SFPQ
**4KI**	4Ki5	2ki5Dc3-ChAT.tif	SFPQ	sALS	4Ki5Dc3	Dc3	Dc	4Ki5	SFPQ
**4KI**	4Ki5	2ki5Dl1-ChAT.tif	SFPQ	sALS	4Ki5Dl1	Dl1	Dl	4Ki5	SFPQ
**4KI**	4Ki5	2ki5Dr1-ChAT.tif	SFPQ	sALS	4Ki5Dr1	Dr1	Dr	4Ki5	SFPQ
**4KI**	4Ki5	2ki5Dr2-ChAT.tif	SFPQ	sALS	4Ki5Dr2	Dr2	Dr	4Ki5	SFPQ
**4KI**	4Ki5	2ki5Dr3-ChAT.tif	SFPQ	sALS	4Ki5Dr3	Dr3	Dr	4Ki5	SFPQ

**4KI**	4Ki5	2ki5Dc1	SFPQ	sALS	4Ki5Dc1	Dc1	Dc	4Ki5	SFPQ
**4KI**	4Ki5	2ki5Dc2	SFPQ	sALS	4Ki5Dc2	Dc2	Dc	4Ki5	SFPQ
**4KI**	4Ki6	2ki6DR2-ChAT.tif	SFPQ	sALS	4Ki6DR2	DR2	DR	4Ki6	SFPQ
**4KI**	4Ki6	2ki6DR3-ChAT.tif	SFPQ	sALS	4Ki6DR3	DR3	DR	4Ki6	SFPQ
**4KI**	4Ki6	2ki6DR4-ChAT.tif	SFPQ	sALS	4Ki6DR4	DR4	DR	4Ki6	SFPQ
**4KI**	4Ki6	2ki6DR5-ChAT.tif	SFPQ	sALS	4Ki6DR5	DR5	DR	4Ki6	SFPQ
**4KI**	4Ki6	2ki6DR1	SFPQ	sALS	4Ki6DR1	DR1	DR	4Ki6	SFPQ

Table S2 | List of images used for FUS and SFPQ cellular localisation in (Tyzack *et al*, 2019; Luisier *et al*, 2018); human data.

annotation_patients

Batch	ID	SAMPLE	AGE	SEX	disease_duration_month	CAUSE_OF_DEATH	time_to_death_hours
**4KI**	4Ki1	NC Lumbar SC G & W Tv	71	M	NA	Burst aortic aneurysm	25
**4KI**	4Ki2	NC Lumbar SC G & W Tv	68	F	NA	Colo-rectal metastatic tumour	23
**4KI**	4Ki3	NC Lumbar SC G & W Tv	68	M	NA	Heart disease	40
**4KI**	4Ki4	MND Lumbar SC G & W Tv	69	M	12	MND & stage 2 respiratory failure	19
**4KI**	4Ki5	MND Lumbar SC G & W Tv	61	M	24	MND	29
**4KI**	4Ki6	MND Lumbar SC G & W Tv	74	F	24	MND	27
**1TG**	1TG1	NC Lumbar SC G & W Tv	70	M	NA	Bronchopneumonia, and heart failure (ishaemia and left ventricular failure)	53
**1TG**	1TG2	NC Lumbar SC G & W Tv	77	M	NA	Pulmonary fibrosis	32
**1TG**	1TG3	MND Lumbar SC G & W Tv	70	M	15	MND	40
**1TG**	1TG4	MND Lumbar SC G & W Tv	81	F	36	MND	33
**1TG**	1TG5	MND Lumbar SC G & W Tv	65	F	23	MND	30
**2TG**	2TG1	NC Lumbar SC G & W Tv	68	M	NA	Colon cancer	25
**2TG**	2TG2	NC Lumbar SC G & W Tv	59	M	NA	Renal failure	15
**2TG**	2TG3	NC Lumbar SC G & W Tv	79	F	NA	Renal failure	21
**2TG**	2TG4	MND Lumbar SC G & W Tv	70	F	29	MND	26
**2TG**	2TG5	MND Lumbar SC G & W Tv	58	M	16	MND	49
**2TG**	2TG6	MND Lumbar SC G & W Tv	69	M	12	MND	19
**3TG**	3TG1	NC Lumbar SC G & W Tv	80	F	NA	Pulmonary embolism	24
**3TG**	3TG2	NC Lumbar SC G & W Tv	81	M	NA	Lung carcinoma; bilateral bronchopneumonia	10
**3TG**	3TG3	NC Lumbar SC G & W Tv	67	F	NA	Bronchopneumonia	34
**3TG**	3TG4	MND Lumbar SC G & W Tv	66	F	12	MND	15
**3TG**	3TG5	MND Lumbar SC G & W Tv	77	F	24	Bronchopneumonia; MND	34
**3TG**	3TG6	MND Lumbar SC G & W Tv	80	F	36	MND	12

Table S3 | Description of the donors from which PMTs were obtained.
